# A Review of Cellularization Strategies for Tissue Engineering of Whole Organs

**DOI:** 10.3389/fbioe.2015.00043

**Published:** 2015-03-30

**Authors:** Michelle E. Scarritt, Nicholas C. Pashos, Bruce A. Bunnell

**Affiliations:** ^1^Center for Stem Cell Research and Regenerative Medicine, Tulane University School of Medicine, New Orleans, LA, USA; ^2^Bioinnovation PhD Program, Tulane University, New Orleans, LA, USA; ^3^Department of Pharmacology, Tulane University School of Medicine, New Orleans, LA, USA

**Keywords:** tissue engineering, native scaffolds, cellularization, whole organs, matrices, recellularization, decellularization

## Abstract

With the advent of whole organ decellularization, extracellular matrix scaffolds suitable for organ engineering were generated from numerous tissues, including the heart, lung, liver, kidney, and pancreas, for use as alternatives to traditional organ transplantation. Biomedical researchers now face the challenge of adequately and efficiently recellularizing these organ scaffolds. Herein, an overview of whole organ decellularization and a thorough review of the current literature for whole organ recellularization are presented. The cell types, delivery methods, and bioreactors employed for recellularization are discussed along with commercial and clinical considerations, such as immunogenicity, biocompatibility, and Food and Drug Administartion regulation.

## Introduction

An effective alternative to traditional organ transplantation is needed in order to increase the number of organs available for transplantation, decrease patient wait-list times, and improve long-term outcomes. In an effort to address this need, research focused on whole organ tissue engineering has flourished over the last 5 years. Since the first reports of skin tissue engineering over 30 years ago, tremendous progress has been made in engineering tissues such as skin, cartilage, or bladder (Hall et al., 1966; Spira et al., 1969; Burke et al., 1981; O’Connor et al., 1981). However, the engineering of whole organs has been impeded by the lack of an adequate scaffold. Thus, the recent surge of research in whole organ engineering can also be attributed to the implementation of a technique known as decellularization to whole organs.

Decellularization removes cells from the extracellular matrix (ECM) of a tissue to produce a three-dimensional organ scaffold (Gilbert et al., 2006; Badylak et al., 2009, 2011; Crapo et al., 2011). Decellularization of “simple” tissues and small organ biopsies was initially reported in the late 80s (Lwebuga-Mukasa et al., 1986). Nearly two decades later, decellularization was successfully adapted for the generation of a whole-heart scaffold (Ott et al., 2008). In the subsequent years, intact lungs, livers, kidneys, and pancreas from rodents, pigs, primates, and humans have been decellularized using similar approaches (Ross et al., 2009; Ott et al., 2010; Petersen et al., 2010; Price et al., 2010; Shupe et al., 2010; Uygun et al., 2010; Baptista et al., 2011; Barakat et al., 2012; Bonvillain et al., 2012; Orlando et al., 2012, 2013; Sullivan et al., 2012; Goh et al., 2013; Mirmalek-Sani et al., 2013a; Song et al., 2013). Prior to this development, creation of a full-scale scaffold with the intricate architecture and composition needed to engineer functional organs was very challenging.

Decellularized scaffolds have been seeded with various cell types, which has resulted in reports of tissue-specific functionality *in vitro* as well as *in vivo* after short-term transplantation into animal models (Ott et al., 2008, 2010; Petersen et al., 2010; Uygun et al., 2010; Bao et al., 2011; Song et al., 2013; Jiang et al., 2014; Kadota et al., 2014; Robertson et al., 2014). Despite remarkable progress, significant challenges still exist, namely scaling up techniques to human-sized organs, finding clinically relevant cell types for recellularization, and completely rebuilding the vasculature and parenchyma of organ scaffolds for long-term function post-transplantation.

The aim of this review is to provide an overview of the recent progress and emerging challenges in whole organ engineering.

## Decellularization for Generation of Organ Scaffolds

### Decellularized organ matrices: What’s left behind?

#### Defining decellularization

Decellularization employs detergents, salts, enzymes, and/or physical means to remove cells from tissues or organs while preserving the ECM composition, architecture, bioactivity, and mechanics. A plethora of decellularization methods exist for different applications [reviewed in (Gilbert et al. (2006), Badylak et al. (2011), and Gilbert (2012)]. Because variation in decellularization methods obscures data comparisons, determining an optimal decellularization method is somewhat enigmatic. Nevertheless, with an ever growing list of new publications, the feasibility of whole organ decellularization is indisputable.

The key criteria for comparison of decellularization methods are the efficiency of cell removal and the adequacy of ECM retention. Crapo et al. recommended that removal of cells be evaluated visually via DAPI or hematoxylin and eosin (H&E) staining coupled with quantification and gel electrophoresis. The goal is to have <50 ng dsDNA/mg tissue (dry weight) remaining after decellularization; in addition, the fragment length of the DNA should be <200 bp (Crapo et al., 2011). Adherence to these guidelines should help reduce the immunogenicity of scaffolds and render them suitable for clinical application.

#### The effect of decellularization on ECM composition

In regards to ECM retention after decellularization, evaluation of the composition, structure, and mechanics of organ scaffolds is critical. Maintenance of the architecture and composition of the ECM is the greatest benefit of decellularized whole organ scaffolds; however, it is also one of the main challenges. Although many groups have demonstrated retention of collagen, laminin, elastin, and fibronectin after decellularization, reduction or depletion of ECM proteins and growth factors has also been reported (Akhyari et al., 2011; Petersen et al., 2012; Wallis et al., 2012; Ren et al., 2013; Caralt et al., 2015). Petersen et al. (2012) reported that lung decellularization methods differentially affect ECM proteins; sodium dodecyl sulfate (SDS) depleted elastin and collagen to a greater degree than decellularization using CHAPS detergent, but both detergents substantially reduce glycosaminoglycan content. Comparing four rat heart decellularization protocols, Akhyari et al. (2011) concluded that none of the protocols were ideal for generating intact scaffolds. They found that if a protocol led to better preservation of ECM proteins, it largely failed to remove cell debris. Conversely, when cell debris was adequately reduced, retention of ECM proteins suffered. Similar results have been reported for optimization of kidney decellularization (Caralt et al., 2015). Although kidneys decellularized using Triton X-100 retained growth factors and ECM components, cells were not adequately removed; whereas, decellularization with SDS was able to sufficiently remove cells while preserving the ECM (Nakayama et al., 2010, 2011; Orlando et al., 2012; Sullivan et al., 2012; Caralt et al., 2015). Therefore, striking a balance between cell removal and ECM preservation is vital to deriving the optimal decellularization protocol. It is important to note that the optimal procedure may be different for each organ due to their unique anatomy.

#### The effect of decellularization on ECM structure

The retention of major ECM components, such as collagen and laminin, lends to preservation of the ultrastructure of the scaffold, which may facilitate recellularization by providing spatial orientation. Corrosive casting has been used to demonstrate that important parenchymal structures, such as the bile duct of rat livers and the bronchial tree and alveoli of rat lungs, are intact after decellularization (Soto-Gutierrez et al., 2011; Kajbafzadeh et al., 2014). For heart scaffolds, heterotopic implantation demonstrated that the tricuspid valve was competent while scanning electron microscopy (SEM) showed retention of myocardial and epicardial fibers (Ott et al., 2008). SEM was also used to demonstrate that the glomerular infrastructure of the kidney and the duct system of the pancreas is intact after decellularization (Goh et al., 2013; Orlando et al., 2013).

In addition to the parenchymal structures, the maintenance of an intact microvasculature is critical for subsequent recellularization of organ scaffolds. It has been shown by micro-CT, perfusion of dyes or microbeads, angiography, and corrosion casting that the structure of the vascular tree is intact after decellularization of hearts, livers, kidneys, lungs, and pancreas (Ott et al., 2008; Petersen et al., 2010; Uygun et al., 2010; Baptista et al., 2011; Barakat et al., 2012; Orlando et al., 2012; Sullivan et al., 2012; Goh et al., 2013; Mirmalek-Sani et al., 2013a; Scarritt et al., 2014; Caralt et al., 2015). Preservation and subsequent reconstitution of the microvasculature and capillary beds will be a crucial aspect to successful recellularization.

In this regard, there have been conflicting reports that reflect the variability of different decellularization techniques. Decellularization of rat livers using 0.5 or 1% Triton X-100 preserved the hierarchical vascular structure; in contrast, 1% SDS led to nearly complete collapse of the vascular network (Shirakigawa et al., 2012). However, rat lungs decellularized using SDS and Triton X-100 failed to maintain capillary integrity while decellularization using sulfobetaine-10/16 amphoteric detergent and Triton X-200 had significantly less extravasation from the vasculature than perfusion-decellularized lungs (Nagao et al., 2013).

In addition to the detergent used, the delivery method also impacts the microvasculature. Constant pressure-based decellularization, but not constant flow, maintained the vascular integrity of rat lung scaffolds (Guyette et al., 2014). To this end, some groups employ gravity-based or pressure-controlled decellularization (Ross et al., 2009; Guyette et al., 2014; Scarritt et al., 2014).

#### The effect of decellularization on scaffold mechanics

Scaffold mechanics are contingent upon ECM composition and structure. Therefore, as an additional evaluation of ECM retention, many researchers have employed the use of traditional engineering techniques such as atomic force microscopy (AFM) and uni- or bi-axial mechanical testing to assess the biophysical properties of organ scaffolds. In many cases, decellularization affects matrix stiffness due to removal of cells and damage to ECM components. After perfusion-decellularization of mouse pancreas, the Young’s elastic modulus was three times greater than native pancreas, indicating stiffening (Goh et al., 2013). In contrast, decellularization of porcine kidney slices decreased stiffness (Nakayama et al., 2010). For heart tissue engineering, bi-axial mechanical testing revealed that the tangential modulus was increased longitudinally and circumferentially after decellularization of rat ventricles (Ott et al., 2008). Bi-axial testing of decellularized porcine hearts also showed increased stiffness (Wang et al., 2010). In contrast, ball burst testing of decellularized porcine ventricle indicated that the maximum force, as well as the extension at maximum force, was not statistically different than native hearts (Wainwright et al., 2010). For lung tissue engineering, ventilation is dependent on appropriate lung compliance and elastance. Thus, the mechanical performance of lung scaffolds has been evaluated using pressure–volume curves (Ott et al., 2010; Petersen et al., 2010; Price et al., 2010). Although decellularized lung scaffolds displayed hysteresis, they also displayed decreased compliance, which was attributed to the removal of surfactant and the depletion of elastin (Daly et al., 2011).

Changes in scaffold stiffness can affect cell fate, particularly in stem cells that can differentiate based on mechanotransduction (Engler et al., 2006, 2007; Reilly and Engler, 2010; Pennesi et al., 2011; Tse and Engler, 2011). Mechanical conditions closest to physiological values will likely direct or maintain the differentiation state of cells. When evaluating stem cell differentiation, matrices with an elastic modulus similar to brain (~1 kPa) were neurogenic while matrices mimicking muscle (10 kPa) or bone (100 kPa) were myogenic or osteogenic, respectively (Engler et al., 2006). Thus, the outcome of organ recellularization can be influenced by the mechanical environment sensed by seeded cells.

## Recellularization of the Parenchyma of Organ Scaffolds

Since the pivotal publication by Ott et al. (2008) demonstrating decellularization of a rat heart, whole organ bioengineering has acquired the attention of scientists and lay-people alike with the goal of creating patient-specific, transplantable organs. Derivation of scaffolds that retain an organ’s three-dimensional structure and composition was the first step, but the task of effective recellularization remains. Recellularization requires appropriate cell sources, an optimal seeding method, and a physiologically relevant culture method, undoubtedly to be accomplished using a bioreactor. For complete organ regeneration, the parenchyma, vasculature, and support components must be reestablished prior to implantation. Many groups have already begun to evaluate techniques and have provided encouraging headway to developing optimal recellularization strategies. However, the technology required for whole organ culture has limited widespread investigation due to complexity, specialization, and in some cases cost. Recellularization studies that encompass implantation *in vivo* are limited. Regardless, high-throughput studies of cell seeding have provided invaluable insight. Tables S1–S5 in Supplementary Material provide a comprehensive overview of the organ recellularization literature covering scaffold source, decellularization method, cell types used for seeding, seeding method, culture method, additional cues, and recellularization outcomes.

### Cell types for parenchymal recellularization

#### Fetal and adult cells

**Figure 1:**
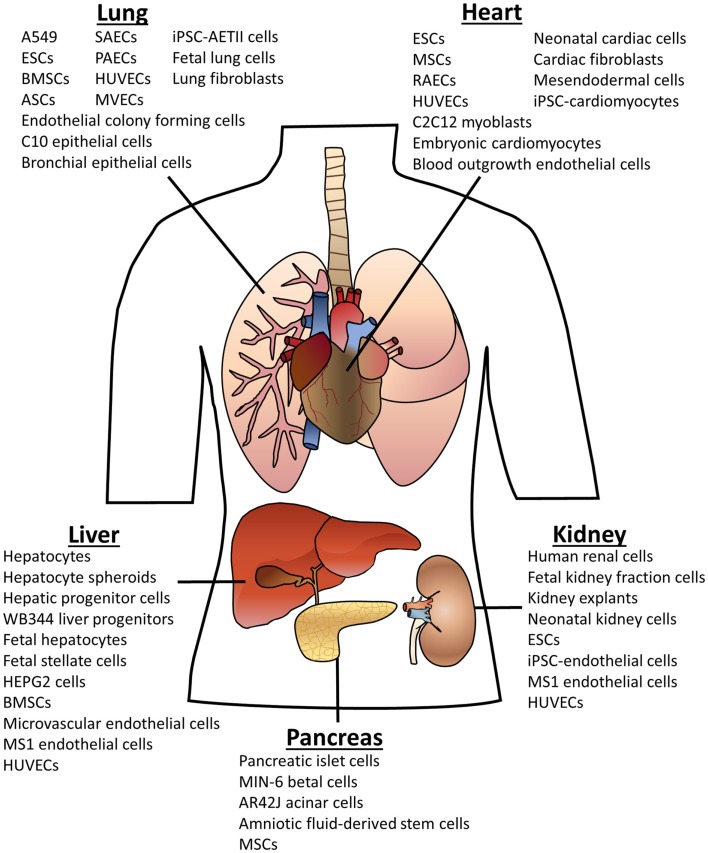
**Cell types used for organ scaffold recellularization**. The cells listed above have been reportedly used for recellularization of the specified organ. Abbreviations: embryonic stem cells (ESCs); bone marrow-derived stem cells (BMSCs); adipose-derived stem cells (ASCs); mesenchymal stem cells (MSCs); induced pluripotent stem cells (iPSCs); human umbilical vein endothelial cells (HUVECs); small airway epithelial cells (SAECs); pulmonary alveolar epithelial cells (PAECs); microvascular endothelial cells (MVECs); alveolar epithelial type II cells (AETII).

As can be seen in Figure 1, many different cell types have been used for organ recellularization. A common theme in the organ bioengineering literature is the use of fetal cells derived from the organ of interest. In general, when these cells are seeded into scaffolds, they retain their phenotypic markers and often display functionality as well as relevant spatial or compartmental orientation (see Tables S1–S5 in Supplementary Material for more details). Indeed, rat lung scaffolds seeded with neonatal or fetal rat lung cells participated in gas exchange after implantation (Ott et al., 2010; Petersen et al., 2010). Neonatal renal cells showed similar success when seeded rat kidneys produce urine *in vivo* (Song et al., 2013). Multiple groups have used fetal hepatic cells for liver recellularization with promising outcomes including urea and albumin production (Baptista et al., 2011; Zhou et al., 2011; Barakat et al., 2012; Sabetkish et al., 2014; Wang et al., 2014). Fetal cells provide proof-of-concept but, nevertheless, are not viable cell types for clinically relevant organ engineering.

Primary adult cells have also been evaluated for recellularization. For example, human renal cells have been utilized for seeding porcine kidney scaffolds, and human alveolar epithelial cells, human lung fibroblasts, and human small airway epithelial cells have been evaluated for recellularization of human or porcine lung scaffolds (Sullivan et al., 2012; Nichols et al., 2013; O’Neill et al., 2013). Although patient-derived primary cells can be obtained from an organ biopsy or a donor organ, primary cells have restricted proliferative capacities and can be difficult to expand for repopulation of a human-sized organ scaffold.

Hepatocytes, for example, are ideal for liver bioengineering, but the availability of human hepatocytes is limited to discarded cadaveric livers (Palakkan et al., 2013). To compound the problem, culture of primary hepatocytes leads to a deterioration in function and stability (Walldorf et al., 2004). Regardless, primary hepatocytes have been evaluated for liver recellularization with mostly positive outcomes, which suggests that organ scaffold culture allows hepatocytes to persist and proliferate better than traditional culture (Uygun et al., 2010; Bao et al., 2011; Baptista et al., 2011; Soto-Gutierrez et al., 2011; Zhou et al., 2011; Barakat et al., 2012; Shirakigawa et al., 2013; Yagi et al., 2013). With primary rat hepatocytes, Uygun et al. (2010) noted over 95% of cells engrafted, and <20% were apoptotic during initial culture. Multiple groups saw similar percentages of engraftment with varying levels of proliferation and apoptosis (Baptista et al., 2011; Soto-Gutierrez et al., 2011; Barakat et al., 2012; Yagi et al., 2013). In addition, hepatocyte-seeded scaffolds have been transplanted by various groups. Bao et al. (2011) transplanted recellularized liver into rats with 90% hepatectomy and reported survival for up to 72 h. After heterotopic transplantation, Uygun et al. (2010) reported that perfusion with minimal ischemic damage to seeded hepatocytes was observed for up to 8 h; *in vitro* albumin, urea, and bile functions were reported as well.

#### Embryonic stem cells

Keeping in mind the ultimate goal of producing a patient-specific organ, investigators have turned to stem or progenitor cells as a source for recellularization. Stem and progenitor cells are of interest because they are easily expanded in culture and able to differentiate into multiple lineages. Of the papers reviewed in Tables S1–S5 in Supplementary Material, embryonic and mesenchymal stem cells (ESCs and MSCs, respectively) were the most prevalent stem cell types used for recellularization. ESCs were often used to assess the effect of organ matrices on differentiation. Decellularized rat kidney scaffolds were seeded with mouse ESCs without any exogenous pro-differentiation signals (Ross et al., 2009, 2012; Bonandrini et al., 2014). Upon seeding, ESCs expressed renal and endothelial markers while pluripotency decreased. On the other hand, Nakayama et al. (2013) seeded human ESCs onto rhesus macaque lung and kidney scaffold slices. Although the ESCs expressed kidney and lung markers, expression was not specific to the organ. Mouse ESC differentiation was also assessed on lung scaffolds in comparison to Gelfoam, Matrigel, and collagen I hydrogel matrix (Cortiella et al., 2010). Based on the expression of lung markers, Cortiella et al. demonstrated that lung scaffolds facilitated differentiation of ESCs into epithelial and endothelial lineages.

Organ ECM is likely limited in its ability to provide all of the necessary signals required for differentiation of ESCs. This limitation may explain why Nakayama et al. did not observe an organ-specific effect of kidney versus lung scaffolds on ESC differentiation. This idea led Ng et al. (2011) to theorize that pre-differentiation of ESC could enhance organ-specific differentiation. Human ESCs were compared to ESCs differentiated into mesendodermal cells (MECs) for recellularization of mouse heart scaffolds. Both cell types lost expression of stem markers and gained expression of cardiac markers, but only ESCs expressed myosin heavy chain 6 while only MECs expressed myosin light chain 2 and 7. After subcutaneous implantation in immunodeficient mice for up to 6 weeks, MEC-seeded scaffolds contained more cells and were better vascularized than ESC-seeded scaffolds. Although the role of organ scaffolds in ESC differentiation is still unclear, scaffolds are likely better suited for enhancing ESC differentiation, when coupled with the appropriate differentiation cocktails, rather than differentiation media alone.

#### Mesenchymal stem cells

Mesenchymal stem cells hold great promise for organ engineering due to their clinical applicability (Gimble et al., 2012). MSCs can be isolated from a patient, from sources such as bone marrow or adipose tissue, and expanded in culture to physiologically relevant numbers. In addition to their ability to differentiate into various cell types, MSCs are involved in tissue repair and can provide stromal support through the secretion of cytokines and chemokines, a role that is likely to facilitate better integration of seeded organs upon transplantation (Toma et al., 2002; Wang et al., 2005; Qian et al., 2008). Interestingly, there is evidence that organ scaffolds enhance the differentiation of MSCs (Jiang et al., 2014). Jiang et al. statically cultured mouse bone marrow-derived MSCs on mouse liver scaffolds in the presence of hepatic differentiation media for 4 weeks. The three-dimensional liver scaffold significantly enhanced MSC differentiation into hepatocytes in comparison to traditional 2D cell culture. In addition, seeded liver scaffold sections rescued liver function after transplantation into a mouse model of fulminant hepatic failure. Ji et al. (2012) took a slightly different approach by harvesting MSC-derived hepatocytes from liver scaffolds and implanting them into mice with CCl_4_-induced fibrotic livers. The cells were able to increase survival, improve liver function, and decrease collagen deposition to restore liver architecture. Heart scaffolds were also able to enhance MSC differentiation into cardiomyocytes, particularly when coupled with mechanical and electrical stimulation (Wang et al., 2013).

The ability of the scaffold to promote MSC differentiation is likely due to organ-specific cell–ECM communications (Guilak et al., 2009; Zhang et al., 2009; Reilly and Engler, 2010). Shamis et al. (2011) determined that the organized, organ-specific stroma of lung or liver matrices was required for localized cell differentiation of hepatocytes and alveolar cells. Both human adipose- and bone marrow-derived MSCs (ASCs and BMSCs, respectively) differentiated toward pulmonary epithelial lineages when seeded into decellularized rat lungs (Mendez et al., 2014). Mendez and colleagues reported that both ASCs and BMSCs expressed pro-surfactant protein C (alveolar type II cell marker), but only ASCs expressed Clara cell secretory protein (club cell marker), and the BMSCs expressed cytokeratin-5 (basal epithelial cell marker). ASCs also showed a propensity for attaching in the upper airways of lung scaffolds, an ability that was not demonstrated by BMSCs, which suggests that these two types of MSCs may have unique attachment properties. Several other groups have seeded lung scaffolds with MSCs and demonstrated attachment and persistence in culture (Daly et al., 2011; Nichols et al., 2013; O’Neill et al., 2013). Indeed, MSCs have been shown to attach to lung scaffolds regardless of decellularization method, sterilization method, storage time, or disease state suggesting that MSCs have a wide-range of application in recellularization (Booth et al., 2012; Wallis et al., 2012; Bonenfant et al., 2013; Sokocevic et al., 2013; Scarritt et al., 2014; Wagner et al., 2014).

Stem cells from amniotic fluid, cord blood, placenta, amnion, olfactory mucosa, and Wharton’s jelly may also have application in organ engineering. For example, umbilical cord blood-derived progenitor cells differentiated into respiratory epithelial cells (Berger et al., 2006). Recently, human amniotic fluid derived stem cells were seeded onto porcine pancreas scaffolds and attached, migrated, and expanded throughout the scaffold (Mirmalek-Sani et al., 2013a). Oberwallner et al. (2014) evaluated human umbilical cord blood MSCs for seeding sections of human heart scaffolds. These MSCs infiltrated the matrix and displayed increased viability and metabolism when grown in the presence of the scaffold. Thus, these alternative sources of stem cells may also be of value to organ recellularization.

Organ-specific stem cells, although not yet applied to organ engineering, would provide a physiologically relevant cell source for recellularization. For example, lung-resident MSCs have been identified in mice and humans that have the ability to differentiate into club cells and alveolar type I and type II cells (Lama et al., 2007; Hoffman et al., 2011; Wagner et al., 2013).

#### Induced pluripotent stem cells

Another promising cell type for organ recellularization is induced pluripotent stem cells (iPSCs). iPSCs are generated by reprograming somatic cells using specific pluripotent genes that render an “embryonic-like” state (Yu et al., 2007). This approach could facilitate the use of patient-derived cells to provide a cell source for the construction of patient-specific organs. Recent investigations have harnessed iPSCs for lung recellularization (Ghaedi et al., 2013). Human iPSCs were differentiated to alveolar epithelial type II cells (AETII) with high efficiency. Cells with an AETII phenotype expressed typical lung markers including surfactant protein C and B. When these cells were seeded into rat and human lung matrices, they readily adhered, proliferated, and maintained expression of alveolar markers. Additionally, AETII cells could be induced to differentiate into an AETI phenotype. For recellularization of mouse heart matrices, iPSC-derived myocyte progenitors were delivered to the scaffold via the aorta (Lu et al., 2013). The cells repopulated the matrix and began spontaneous contraction after 20 days; however, echocardiogram revealed an irregular wave morphology suggesting that the cells lacked an organized conduction system. Regardless, the endocardium harbored muscle-like and vessel-like structures formed by seeded iPSC-myocytes. The resulting tissue was shown to be drug responsive to an adrenergic agonist as well as an inducer of ventricular arrhythmias. Conversely, Oberwallner et al. (2014) compared heart recellularization with iPSC-derived cardiomyocytes to MSCs and found that iPSC-cardiomyocytes attached less fervently than MSCs even if fibronectin was added to scaffolds to enhance adhesion. Thus, the use of iPSCs for organ recellularization requires additional investigation.

#### Support cells

The integration of mural, stromal, immune, and interstitial cell types is also of utmost importance to complete recellularization in order to generate an organ that can renew and respond to injury. Fibroblasts, for example, may have a significant role in matrix remodeling in organ scaffolds. In addition, fibroblasts have been shown to enhance AETII cell function (Adamson, 1992; Griffin et al., 1993). Immune cells such as Kupffer cells for the liver or alveolar macrophages may enhance recellularization. Pericytes and nerve cells may also be important contributors to complete recellularization. A report by Barakat et al. (2012) used human fetal stellate cells, pericytes that surround the sinusoids of the liver, to enhance the attachment and viability of human fetal hepatocytes seeded into porcine liver scaffold segments. To enhance hepatocyte recellularization of rat liver scaffolds, Kadota et al. (2014) co-seeded BMSCs as a supportive cell type. The inclusion of the BMSCs enhanced hepatocyte integration into the scaffold by facilitating migration into the parenchymal space where hepatocytes aligned in cords. The BMSCs were principally located in the portal area and intraluminal surface of vessels suggesting a different niche or binding affinity than the hepatocytes. Not only does this demonstrate the value of incorporating support cells into recellularization strategies but also the need for multiple cell types for complete coverage of organ scaffolds.

### Parenchymal recellularization: Whole organ seeding methods

Although some of the same cell types have been evaluated for recellularization of different organs, the seeding method is highly dependent on the organ itself. A vascular tree is common to all organs, but additional non-vascular routes can be of use for seeding. The delivery method is also of import to cell seeding due to the mechanical influence of fluid shear stress and pressure. Seeding methods for each organ are presented. Tables S1–S5 in Supplementary Material include publications describing the technical details of seeding methods for each organ. These investigations provide critical clues toward generating optimal seeding strategies.

#### Liver

The liver can be seeded from multiple vascular routes, namely the portal vein, the hepatic artery, or the inferior vena cava (IVC) (hepatic veins). Most groups use either the portal vein or the vena cava (Wang et al., 2014); however, employing the use of multiple routes very likely facilitates the spatial arrangement of cells. Indeed, perfusion seeding of liver scaffolds via the vena cava deposited cells in the pericentral area, while seeding via the portal vein deposited cells in the periportal area (Baptista et al., 2011). Interestingly, Baptista et al. also noted that the directionality of flow during perfusion affected the alignment of cells.

Another group found that seeding via the portal vein or hepatic vein was not as effective as seeding hepatocytes, suspended in collagen gel, directly into the liver using a needle (Shirakigawa et al., 2013). Direct injection of cells, however, may lead to cell aggregation, poor cell engraftment, and insufficient distribution throughout the scaffold. A study by Soto-Gutierrez et al. (2011) demonstrated this by evaluation of different methods to recellularize the liver parenchyma of rat scaffolds with mouse hepatocytes. Five injections into the hepatic lobes using a needle generated <13% engraftment of seeded cells. Perfusion seeding, on the other hand, led to more favorable outcomes. Cells introduced to the liver by continuous media perfusion within a bioreactor resulted in roughly 70% cell engraftment, while approximately 86% engraftment was achieved when infusing cells directly into the liver perfusion circuit in 10–15 min steps. This “step-wise” method was also successfully employed (with >95% engraftment) by Uygun et al. (2010) when seeding primary rat hepatocytes into rat liver scaffolds, by Yagi et al. (2013) when seeding primary porcine hepatocytes into porcine liver scaffolds, and by Jiang et al. (2014) when seeding mouse BMSCs into mouse scaffolds. Multiple infusions will likely be necessary to deliver an adequate number of cells to reconstitute the liver sufficiently. Indeed, multi-step serial infusion was used to increase the number of seeded cells from 50 to 200 million (representing 20% of a rat’s liver mass) (Uygun et al., 2010). To account for 5–10% of the liver mass of a human lung, 10 billion hepatocytes would be required (Caralt et al., 2014).

Overall, perfusion seeding is most efficient at distributing cells throughout the liver scaffold, and future investigations would likely benefit from utilizing multiple seeding routes. In addition, the use of the hepatic duct system for seeding has not yet been investigated.

#### Kidney

The kidney can be seeded via the vasculature or the ureter; however, antegrade perfusion through the renal artery is the most common route in the kidney recellularization literature. Bonandrini et al. (2014) seeded mouse ESCs at 0.2 mL/min through the renal artery and reported an even distribution of cells with over 97% cell attachment. Caralt et al. (2015) reported that renal arterial perfusion seeding of 40 million human renal cortical tubular epithelial (RCTE) cells at 25 mL/min led to about 50% coverage of the renal area.

Two groups utilized the ureter for seeding (Ross et al., 2009; Song et al., 2013). In static culture experiments, Ross and colleagues saw better distribution and retention of cells when seeding through the renal artery than through the ureter (>95% retained versus ~50% retained). However, Song et al. (2013) noted site-specific adhesion of neonatal rat kidney cells as well as polarity when seeding through the ureter. Kidney scaffolds seeded with neonatal kidney cells and human umbilical vein endothelial cells (HUVECs) could produce urine *in vitro* and *in vivo*.

Song et al. enhanced seeding through the ureter by generating a pressure gradient via a vacuum. This may account for the discrepancy in the observations of ureter-based seeding reported by Ross et al. in comparison to Song et al. Another important observation of Song et al. was that a vacuum pressure exceeding 70 cm H_2_O damaged the parenchymal tissue while a pressure of 40 cm H_2_O was acceptable. This illustrates the importance of the mechanical environment during seeding.

#### Lung

The lung has two seeding routes, the vasculature and the airway. Utilizing the trachea for seeding of fetal rat lung cells and the pulmonary artery for seeding of HUVECs, Ott et al. (2010) demonstrated reconstitution of the alveolar-capillary membrane lending to gas exchange *in vitro* and *in vivo*. Both cell types were seeded by gravity and then cultured in a bioreactor at a set pressure range of 10–15 mmHg. Petersen et al. seeded cells via both routes as well; however, cells were instilled at 3 mL/min into the vasculature or as a bolus into the airway until the lungs were fully inflated (Petersen et al., 2010; Calle et al., 2011). This group also demonstrated gas exchange after implantation of regenerated lungs. Price et al. (2010) on the other hand, seeded cells via the trachea only and utilized ventilation-based organ culture at 180 breaths/min. Trachea-only seeding was also employed by Cortiella et al. (2010) who used a rotating bioreactor for organ culture. After these four initial reports, many other groups adopted similar seeding methods (coupled with static culture) to evaluate different cell types for recellularization (see Table S3 in Supplementary Material for more information).

Ultimately, complete lung recellularization will likely be accomplished by seeding the airway and the vasculature, via both the pulmonary artery and pulmonary veins. The main challenge will be distributing cells throughout the branching structure of the airway and vasculature for full-coverage of the proximal and distal regions of the scaffold.

#### Heart

For heart recellularization, cells can be seeded into the coronary arteries via the aorta. iPSCs delivered via the aorta were able to repopulate a mouse heart matrix (Lu et al., 2013). Because continuous perfusion culture washed out most of the cells, Lu et al. opted to perfuse the heart at 8-h intervals. By the end of the culture period, iPSC-derived cardiomyocytes formed myofilaments, were drug responsive, and spontaneously contracted. Ng et al. (2011) seeded a single bolus of MECs or ESCs into the aorta of mouse heart scaffolds followed by static culture for 2 weeks. Though no beating was observed after subcutaneous implantation, seeded scaffolds were vascularized by the host and cells expressed cardiac and endothelial markers.

Aorta-based seeding of the coronary arteries has promise, but seeding the ventricular wall, on the other hand, is more difficult. Cell engraftment into the ventricular wall was accomplished by direct injection with a needle by multiple groups (Ott et al., 2008; Crawford et al., 2012; Hulsmann et al., 2013; Robertson et al., 2014; Weymann et al., 2014). Ott et al. (2008) conducted five serial injections of neonatal rat cardiac cells into the anterior left ventricle; this was followed by bioreactor culture with 20 mL/min atrial flow and 6 mL/min coronary flow as well as electrical stimulation. Although, dense cellularity was noted at the injection site, cells were not well distributed throughout the scaffold. Weymann et al. (2014) also seeded cells using five injections in the left ventricle of porcine heart scaffolds and reported approximately 50% cellularity at the injection site, but less seeding in the distal portions. Regardless, Weymann et al. observed electrical activity, and Ott et al. (2008) noted the formation of contractile fibers, cardiomyocyte contraction in response to electrical stimuli, pump function, and drug responsiveness.

#### Pancreas

Because pancreas decellularization is less widely studied than other organs, investigation of recellularization is currently limited. The pancreas can be seeded through the pancreatic duct or the vasculature. The vasculature can be accessed via the portal vein, splenic artery, superior mesenteric artery and veins, or superior pancreaticoduodenal artery. Multiple cell infusions via various routes have been efficacious for pancreas recellularization. Goh et al. (2013) seeded MIN-6 pancreatic beta cells into the portal vein and AR42J pancreatic acinar cells via the pancreatic duct by three 1 mL perfusion steps (with a 20 minute static incubation between steps). MIN-6 cells attached in luminal spaces and larger blood vessels while AR42J cells lined the tubular ductal spaces; no co-localization of the two cell types was observed suggesting that dual seeding is beneficial for complete pancreas recellularization.

### Optimization of parenchymal seeding

Coating of organ matrices has been employed as a technique to enhance cell seeding. After Lecht et al. (2014) determined that the integrin-binding profile of mouse ESCs allows adhesion to laminin and fibronectin but not collagen I or IV, they coated rat lung scaffolds with laminin and fibronectin to enhance seeding. Coating was accomplished by instilling lung scaffolds with conditioned media from A549 pulmonary epithelial cells. They observed enhanced binding of mouse ESCs, a 2.3-fold increase in cellularity, and better cell dispersion throughout the scaffold (Lecht et al., 2014). Another group evaluated coating mouse lung scaffolds with Matrigel or collagen I (Jensen et al., 2012). Mouse ESCs seeded into Matrigel-coated or uncoated scaffolds expressed lung markers (pro-SPC and TTF-1) similarly (Jensen et al., 2012). However, mouse ESCs seeded into collagen I-coated matrices exhibited no pro-SPC expression and minimal TTF-1 expression. Thus, coating with collagen I was actually detrimental to the differentiation of mouse ESCs and coating with Matrigel did not enhance differentiation, which may support the findings of Lecht et al. who reported that mouse ESCs do not adhere to collagen I. The lack of lung-specific differentiation may also be due to increased stiffness caused by collagen coating. Indeed, Jensen et al. reported that collagen coating changed the mechanical properties of the matrix; as previously discussed, stem cells can differentiate according to the stiffness of a matrix (Engler et al., 2006, 2007; Pennesi et al., 2011).

In addition to ESCs, multiple groups have investigated the ECM binding preferences of MSCs and have shown that different integrin-binding profiles affect stem cell attachment and differentiation (Daly et al., 2011; Bonvillain et al., 2012; Frith et al., 2012). Thus, it would be valuable to investigate the use of an ECM coating technique to enhance MSC seeding of organ scaffolds.

Optimal cell density and cell number will need to be determined for seeding. Increasing the cell density increases the possibility of the formation of cell aggregates that could occlude vessels and cause necrosis by impeding nutrient transfer. The use of EDTA to prevent clusters has been suggested; however, the use of a cell strainer can also reduce cell aggregates prior to seeding (Calle et al., 2011). Excessively high cell numbers may also lead to vessel occlusion. Thus, using low cell concentrations or multiple cell infusions rather than a large, concentrated bolus of cells may be beneficial. Indeed, multi-step cell infusions enhanced recellularization of livers (Uygun et al., 2010; Soto-Gutierrez et al., 2011).

For the seeding of multiple cell types, many variables come into play. Multiple cells can be seeded sequentially in the same route or simultaneously in the same cell suspension. Indeed, Kadota et al. (2014) found that simultaneous seeding of hepatocytes and MSCs was superior to sequential seeding.

## Recellularization of the Vasculature of Organ Scaffolds

The clinical success of any tissue-engineered construct is dependent on its survival and integration into the body, particularly by establishing a connection to the blood supply. Because the diffusion limit of oxygen in the human body is 150–200 μm, engineered organs require a vascular network (Folkman and Hochberg, 1973). In addition, the denuded vasculature of organ scaffolds is highly thrombogenic even with anti-coagulation (Baptista et al., 2011; Orlando et al., 2012; Robertson et al., 2014). Therefore, the vasculature of an organ scaffold must be completely recellularized for successful transplantation.

### Cell types for vascular recellularization

#### Endothelial cells

Endothelial cells are the primary cell types used for vascular recellularization. In the first report of organ decellularization and recellularization, rat aortic endothelial cells were used. These cells lined the coronary vessels of rat heart scaffolds lending to decreased thrombogenicity upon implantation (Ott et al., 2008; Robertson et al., 2014). For the re-endothelialization of rat lung scaffolds, Ott et al. (2010) seeded ~66 million HUVECs by gravity perfusion into the pulmonary artery and the pulmonary vein. Engraftment from the pulmonary artery through the vascular tree to the capillaries was observed after 5 days in a perfusion bioreactor culture. Scaffolds that had been seeded with rat fetal lung cells as well as HUVECs were implanted orthotopically into rats for up to 14 days without any bleeding into the airways, although edema was noted (Song et al., 2011). HUVECs were also employed for re-endothelialization of rat kidney scaffolds and were able to line blood vessels throughout the scaffold (Song et al., 2013). Scaffolds seeded with neonatal rat kidney cells and HUVECs were able to be orthotopically implanted without thrombi formation or hemorrhaging. HUVECs have also been used for liver re-endothelialization by seeding through the portal vein with cells seen lining large and small vessels (Baptista et al., 2011; Shirakigawa et al., 2012, 2013).

Microvascular endothelial cells have the potential for vascular recellularization. Cardiac microvascular endothelial cells perfused into the portal vein of rat liver scaffolds lined the vasculature and remained viable throughout bioreactor culture (Uygun et al., 2010). Petersen et al. (2010) seeded lung microvascular endothelial cells into rat lung scaffolds through the pulmonary artery using a perfusion pump (at 3 mL/min). Transmission electron microscopy identified tight junctions between some of the endothelial cells; however, when scaffolds were implanted, thrombi and bleeding into the airways were observed suggesting that revascularization was incomplete.

Circulating endothelial colony forming cells could potentially be isolated from a patient’s blood for clinically relevant re-endothelialization of organ scaffolds. Indeed, endothelial colony forming cells and blood outgrowth endothelial cells adhered and dispersed throughout the vascular compartment of lung and heart scaffolds (Crawford et al., 2012; Wagner et al., 2014).

#### Embryonic stem cell

In addition to recellularization of the parenchyma, stem cells have also been considered for vascular recellularization. Mouse ESCs seeded into the artery of decellularized rat kidneys and cultured without any exogenous growth factors displayed a flattened endothelial-like morphology within vascular structures (Ross et al., 2009). In addition, these cells were positive for endothelial-specific lectin and vascular endothelial growth factor receptor (VEGFR) (Ross et al., 2012). Bonandrini et al. (2014) also seeded murine ESCs into the renal artery of rat kidney scaffolds and observed repopulation of the glomerular and peritubular capillaries. These cells expressed Tie-2 and CD31 endothelial markers as early as 24 h after seeding, and CD31 expression increased after 72 h.

#### Mesenchymal stem cells

Adipose-derived stem cells were able to attach to the vascular matrix in lung scaffolds derived from healthy and hypertensive rats; the cells lined the vasculature and persisted in static culture for 2 weeks (Scarritt et al., 2014). Although Scarritt et al. did not assess endothelial differentiation, MSCs have been shown to differentiate toward endothelial lineages based solely on their interaction with endothelial ECM (Lozito et al., 2009a,b). In addition, both ASCs and BMSCs have the ability to differentiate into smooth muscle cells, which are a cell type that has not yet been assessed for organ revascularization (Liu et al., 2007; Gong and Niklason, 2011; Marra et al., 2011). The role of smooth muscle in regulating blood pressure and, hence, proper nutrient delivery throughout the body lends to the importance of evaluating this cell type for organ revascularization.

#### Induced pluripotent stem cells

Rat kidney scaffolds were re-endothelialized using human endothelial cells derived from iPSCs (Caralt et al., 2015). The iPSC-endothelial cells lined the branching structures of the vasculature and were found adjacent to the glomeruli. Lu et al. (2013) used a similar approach for recellularization of mouse heart scaffolds by differentiating human iPSCs and human ESCs into multipotent cardiovascular progenitor cells. These cardiovascular progenitor cells were seeded into the matrix via the aorta and then subsequently differentiated into cardiomyocytes, smooth muscle cells, and endothelial cells by perfusion of growth factors. Vessel-like structures were observed in the seeded scaffolds with endothelial cells lining the endocardial surface as well as small coronary arteries. Thus, iPSCs have shown promise for vascular recellularization.

#### Support cells

Another approach that may be of interest for vascular recellularization is the use of the stromal vascular fraction (SVF) from adipose tissue. SVF is a heterogeneous cell mixture containing endothelial cells, mural cells, and immune cells (Gimble et al., 2011; Bourin et al., 2013). The use of a mixed cell population such as the SVF may provide a better environment for engineering competent, renewable vasculature. Indeed, Nunes et al. (2013) generated a functional liver mimic by suspending SVF cells and HepG2 human liver cells within a collagen type I gel. After implantation into mice, the SVF cells assembled into a functional vascular network that could supply the HepG2 cells. Interestingly, SVF cells were often seen in a perivascular location along the newly formed vasculature. In addition, host-derived blood vessels that infiltrated the implanted construct contained SVF cells.

The importance of mural cells to vascular tissue engineering has been demonstrated in simple tissue constructs (Levenberg et al., 2005; Rochon et al., 2010). These cell types may also be beneficial for organ scaffold revascularization. Therefore, future studies should incorporate mural cells such as pericytes and smooth muscle cells to support the endothelium.

### Vascular recellularization: Seeding methods

Seeding of the vascular compartment is commonly accomplished by perfusion through the vascular tree. Most groups employ a perfusion pump, but some use gravity-driven apparatuses for vascular seeding (Ott et al., 2010). Perfusion pressure and flow rate will likely be the most influential parameters for vascular seeding (Lichtenberg et al., 2006).

Although many groups have seeded parenchymal cells via the vasculature, investigation and optimization of revascularization have been limited. A few groups have employed continuous perfusion seeding at relatively low flow rates for seeding endothelial cells. For recellularization of rat kidney scaffolds, HUVECs were perfused into the renal artery at 1 mL/min followed by an overnight static incubation (Song et al., 2013). Cells lined the blood vessels throughout the scaffold preventing leakage upon transplantation. HUVECs seeded into the portal vein at 0.5 mL/min formed a monolayer and decreased leaks upon blood perfusion (Shirakigawa et al., 2012, 2013). Endothelial cells seeded at 3 mL/min into the pulmonary artery dispersed throughout the matrix; however, bleeding into the airways was observed after orthotopic transplantation (Petersen et al., 2010).

To date, most reports seed via a single route; however, seeding the vasculature antegrade and retrograde may benefit complete revascularization. Indeed, gravity-based seeding of HUVECs into the pulmonary artery and pulmonary veins of rat lung scaffolds prevented hemorrhaging and thrombosis after implantation (Ott et al., 2010; Song et al., 2011). Therefore, seeding via the pulmonary artery and veins by Ott et al. was likely better able to cover the entirety of the vascular tree of lung scaffolds than arterial seeding alone used by Petersen et al.

For the re-endothelialization of rat heart scaffolds, three different seeding routes (or combinations of routes) were evaluated (Robertson et al., 2014). Rat aortic endothelial cells were infused into the aorta, the brachiocephalic artery (BA), the inferior vena cava (IVC), or both the BA and the IVC. The dual seeding method resulted in the highest cellularity and a uniform distribution of cells. Seeding via the BA delivered cells mostly to the vasculature of the left ventricle while seeding via the IVC cellularized the vasculature of the right ventricle. When perfused with human thrombin and protein C, seeded endothelial cells exhibited thrombomodulin and thrombin-mediated protein C activity. Heart scaffolds that were re-endothelialized (in combination with seeding of rat neonatal cardiac cells) and implanted heterotopically into rats for 7 days had less clotting than controls, illustrating the importance of revascularization for organ engineering.

Baptista et al. (2011) compared antegrade seeding through the portal vein and retrograde seeding through the vena cava of ferret liver scaffolds. Seeding through the vena cava deposited endothelial cells into the pericentral area while seeding via the portal vein deposited cells in the periportal area. To evaluate dual seeding using both routes, DAPI-staining cells were seeded into the portal vein, and red fluorescent beads were instilled into the vena cava. Baptista and colleagues observed the beads in the center of the liver lobule surrounded by cells in the periportal area. Thus, for liver recellularization, seeding via multiple routes led to better cell distribution and coverage.

Because the vasculature is crucial to the successful integration and survival of an organ transplant, complete revascularization should be of utmost importance moving forward.

### Evaluation and enhancement of vascular recellularization

Transplantation of a regenerated organ with anastomosis to the host blood supply is the ultimate assessment of successful re-endothelialization; however, whole organ implantation studies require technical experience with microsurgery for rodent models and are costly for larger animal models. As a surrogate to transplantation, blood perfusion has been used to evaluate revascularization. Baptista et al. (2011) perfused blood through mouse endothelial cell-seeded ferret liver scaffolds to assess platelet deposition. Re-endothelialized scaffolds displayed significantly less platelet aggregation than unseeded controls. Shirakigawa et al. (2013) perfused heparinized rat blood through HUVEC-seeded liver matrices and reported that, in comparison to acellular matrices, there were not as many leaks. Blood perfusion is a viable preliminary (or pre-implantation) approach to assessing endothelialization, particularly because having an intact vascular barrier is critical to the success of organ transplantation. As illustrated by Robertson et al. (2014), perfusion with human thrombin and protein C is another valuable approach to evaluating thrombomodulin and thrombin-mediated protein C activity of re-endothelialized scaffolds prior to implantation studies.

Because effective re-endothelialization would prevent thrombosis, an interesting approach to enhancing vascular recellularization is “heparin layering” – a technique developed to render scaffolds thromboresistant (Bao et al., 2011). The vascular compartment of rat liver scaffolds was coated with nine layers of heparin using a layer-by-layer self-assembly technique. This technique rendered the scaffold thromboresistant for 3 h of implantation without endothelial cells. However, the application of heparin layering alongside cell seeding is unknown.

Vascularization of organ scaffolds has made considerable progress, but the generation of a competent vasculature for long-term implantation has not yet been demonstrated.

## Bioreactors for Whole Organ Engineering

### Bioreactor parameters and application

For the culture of regenerating organs, a bioreactor is required to deliver nutrients to the core of the tissue via perfusion. This can be accomplished with a very simple apparatus consisting of a perfusion pump and an organ chamber with at least one inlet and one outlet (Bao et al., 2011; Jiang et al., 2014; Sabetkish et al., 2014). On the other hand, more sophisticated equipment has been developed to monitor flow rate, pressure, oxygenation, and other parameters. There are also bioreactors that can not only monitor these conditions but can also manipulate them. For example, “self-correcting” bioreactors that can adjust flow rate based on a max pressure or that can infuse media based on glucose and lactate concentrations have been described (Ott et al., 2008, 2010; Barakat et al., 2012; Ross et al., 2012; Hulsmann et al., 2013).

In addition to perfusion, organ-specific stimuli are also crucial to developing functioning organs. Mechanical cues may help maintain phenotypes or promote differentiation of progenitor cells (Clause et al., 2010; Ismail et al., 2012; Schmitt et al., 2012; Zheng et al., 2012). Bioreactors should generate an environment that is organ-specific and able to mimic *in vivo* conditions.

#### Liver

For liver engineering, the bioreactors described in recent publications commonly employ vascular perfusion without any other mechanical influences. For example, multiple groups employ a simple apparatus consisting of a peristaltic pump, an oxygenator, a bubble trap, and a chamber (without rigid surfaces) for rat and pig liver culture (Uygun et al., 2010; Soto-Gutierrez et al., 2011; Yagi et al., 2013; Kadota et al., 2014). For ferret liver culture, Barakat et al. (2012) also used a perfusion-based system consisting of a housing chamber with a sterile, vented organ cassette modified from a LifePort Kidney Transporter. Using an infusion pump, media was circulated between the cassette and a 3 L reservoir through oxygenator tubing connected to a mixture of air and CO_2_.

For the regeneration of liver (and pancreas), perfusion through the ductal system may be beneficial and has not yet been investigated. In addition, a dual perfusion system for the portal vein and the hepatic artery of the liver may also be beneficial.

#### Kidney

For recellularization of rat kidney scaffolds, Caralt et al. (2015) constructed a perfusion-based system from two adjacent glass flanges with a valve and septum. Kidney scaffolds were seeded in the bioreactor at a high flow rate of 25 mL/min (232 mmHg) by antegrade pulsatile perfusion to the renal artery before decreasing the flow rate to 4 mL/min for culture. Although human RCTE cells were able to seed approximately half of the renal area and form tubular structures, Caralt et al. noted that oxygen access may have been limited in some areas of the scaffold suggesting that further optimization of bioreactor culture is needed.

Bioreactors that facilitate perfusion through both the renal artery and the ureter may assist kidney regeneration. Song et al. (2013) created a system for access to the renal artery as well as the ureter. Their seeding chamber included a port for the withdrawal of air to generate a negative pressure environment and create a transrenal pressure gradient to facilitate seeding through the ureter; however, for subsequent whole organ culture, perfusion alone was utilized while the ureter drained passively into the chamber. Whether or not media perfusion through the ureter would be beneficial to organ culture is unknown.

#### Lung

For lung engineering, ventilation of media or air (mediated by a bioreactor) has been reported (Ott et al., 2010; Petersen et al., 2010). The transition from wet to dry ventilation is likely necessary for successful re-epithelialization of the airway. Calle et al. (2011) built a custom, user-friend, inexpensive bioreactor for lung regeneration that utilizes negative pressure ventilation by withdrawing air from a chamber using a syringe pump. This bioreactor was used by Petersen et al. (2010) for regenerating rat lung tissue for transplantation. Subsequent evaluation of the bioreactor culture system for the long-term maintenance of native lung tissue indicated that this bioreactor could provide sufficient nutrient supply and mechanical stretch to foster cell survival (Petersen et al., 2011). Interestingly, media delivery by ventilation was able to provide nutrients to the parenchyma and vasculature while perfusion alone was not adequate to maintain cells in the parenchyma. For scaling up to larger animal models, this bioreactor design was easily adjusted for use with rhesus macaque lungs (Bonvillain et al., 2013). Conversely, commercial large-organ bioreactors have also been employed for the culture of porcine or human lungs (Nichols et al., 2013; Gilpin et al., 2014).

#### Heart

Heart tissue engineering applications employing both mechanical stretch and electrical stimuli have shown promise in promoting an organized beat function and conduction (Ott et al., 2008; Wang et al., 2013). Ott et al. utilized a bioreactor based on a water-jacketed working heart system from the company Radnoti. This bioreactor incorporated pressure transducers and flow meters to permit the measurement of preload and afterload, as well as inflow and outflow, in order to deliver physiologically relevant intraventricular pressures. In addition, synchronized electrical stimulation could be delivered at 5–20 V for pacing of the heart construct.

Weymann et al. (2014) also used a commercial bioreactor system, the BIOSTAT B-DCU II from Sartorius Stedim Biotech. The system consisted of a control tower connected to a custom glass culture vessel with precise temperature and pH control. Using up to six peristaltic pumps, 5 L of media could be continuously circulated through porcine hearts at a set flow rate and pressure.

Hulsmann et al. (2013) developed a low-cost, modular bioreactor system for whole-heart cultivation that utilized coronary perfusion and three-dimensional mechanical stimulation. Using an operating platform based on LabVIEW^®^, the left ventricle of decellularized rat hearts undergoes controlled stretching using an inflatable latex balloon. Balloon filling was activated by a syringe pump while a membrane pump was used to deliver volume-strokes at set frequencies. In addition, the pressure inside the system was monitored and controlled by a pressure sensor. For perfusion, a pressure transducer and a peristaltic pump drove a tubing system with an incorporated bubble trap. This highly integrated system was augmented with a media reservoir consisting of a double-jacket stirrer vessel with a disk-type stirrer. The reservoir was fitted with a sparging ring for gassing media as well as a pH sensor, a pO_2_ sensor, and a temperature sensor for monitoring media conditions. A custom gas mixing apparatus with a flow controller allowed fully automated conditioning of media with air/O_2_, CO_2_, and N_2_ in response to pH and pO_2_ values. Hülsmann et al. also noted that electrophysiological monitoring and stimulation could easily be integrated into their bioreactor system in the future.

### Optimization of bioreactor culture

Proper bioreactor culture will require optimization. Some of the parameters that should be considered when developing a culture protocol include volume, pressure, flow rate, culture time, and mechanical stimuli. As scaffolds become more and more cellularized, the porosity will decrease and the pressure will increase (Lawrence et al., 2009). Therefore, monitoring pressure and adjusting flow rate throughout culture will be critical to preventing shear stress or mechanical damage to seeded cells. Additionally, the cell types commonly used for recellularization are adhesion-dependent; thus, anoikis may occur if cells recirculate for extended periods of time without attaching to the matrix. Culture time and media turnover will require investigation. Indeed, Caralt et al. (2015) measured glucose and lactate in the media of their bioreactor via a valve for media sampling; based on the concentrations of glucose and lactate, they determined that oxygen was limited within their regenerating kidney. Measurement of oxygen and nutrient turnover may be correlated to cell metabolism and, in turn, cell number. More importantly, monitoring metabolism can provide information regarding potentially hypoxic conditions if perfusion is inadequate. These types of analyses will prove advantageous to assessing the progress of bioreactor culture.

Developing non-invasive, non-destructive technologies for assessing organ recellularization is needed. Imaging techniques, such as surface scanning, have been incorporated into bioreactor systems and may be able to monitor organ size and structure (Hulsmann et al., 2013). Another non-invasive/non-destructive imaging method is the combination of multiphoton microscopy and image correlation spectroscopy to assess mechanical properties during decellularization (Merna et al., 2013). Perhaps, a similar system could be used to monitor organogenesis within a bioreactor. Ultimately, the field of organ engineering would greatly benefit from additional non-invasive techniques for the assessment of the completeness of organ regeneration.

Although organ-specific chemical, electrical, and mechanical stimuli are important to effective recellularization, these biophysical cues will need to be highly regulated. In addition, the abnormal, or perhaps unintentional, mechanical stimuli cells undergo during seeding and culture within the context of a bioreactor are still enigmatic. Where some tissue engineers employ direct inoculation of cells using a syringe or a needle, others utilize automated procedures using perfusion pumps or syringe pumps. To that end, investigators should assess the effect of laminar flow, pressure, and shear stress during seeding and whole organ culture on cell outcomes.

## Clinical and Commercial Considerations

The field of organ engineering is rapidly progressing toward commercialization and clinical application. “Scaling up” decellularization/recellularization to a clinically relevant size has been demonstrated using porcine and human tissue (Sullivan et al., 2012; Nichols et al., 2013; Yagi et al., 2013; Gilpin et al., 2014; Weymann et al., 2014). Therefore, immunogenicity and biocompatibility of organ scaffolds has become a topic of interest.

### Whole organ engineering: Immunogenicity

One of the major drawbacks of traditional organ transplantation is the requirement for life-long immunosuppression. Decellularization provides the opportunity to reduce or eliminate donor DNA and major histocompatibility complex (MHC) antigens. As criteria for successful decellularization, removal of cells and reduction of DNA has been widely demonstrated using different organs, models, and methods. Decellularization of rat lungs was shown to significantly reduce the presence of MHC class I and II molecules and DNA (Petersen et al., 2010). Scaling up to the rhesus macaque primate model, SDS-based decellularization of kidney or lung slices removed MHC class I and class II antigens [also known as human leukocyte antigens (HLA) E and DR] (Nakayama et al., 2013).

To evaluate immunogenicity, implantation of decellularized organ scaffolds has been reported. A segment of decellularized mouse pancreas containing <50 ng/mg dry weight DNA was implanted subcutaneously into mice for 2 weeks; despite mononuclear cell infiltration, the implant did not elicit a foreign-body response or cytotoxicity (Goh et al., 2013). In another report, porcine kidney scaffolds seeded with murine endothelial cells indicated no cytotoxic effect (Orlando et al., 2012); however, orthotopic implantation of the scaffolds into pigs for 2 weeks displayed thrombi formation (due to the presence of a denuded vasculature) as well as infiltration of inflammatory cells (Orlando et al., 2012). In one case, the implant was completely encased in a fibrous capsule indicating a foreign-body response. Thus, more work is necessary to assess immunogenicity of decellularized organ scaffolds, particularly considering the diverse array of decellularization methods in the literature.

### Biocompatibility of porcine scaffolds

Decellularization followed by recellularization using patient-derived cells has the potential to generate a new, patient-specific organ and eliminate the need for immunosuppression. However, in order to increase the pool of organs available for transplantation, scaffold sources cannot be derived from healthy, transplantable organs. One approach is to use animal organs as a scaffold source for human tissue engineering. Ethical issues preclude the use of primate organs for human tissue engineering, so most researchers have turned to pigs. When considering commercial production and clinical use of animal organ scaffolds, pigs are an ideal choice because they are physiologically similar to humans, breed well in captivity, have a relatively short gestation period, and can be raised in sterile conditions.

Decellularization of pig organs is an active area of research with promise as well as challenges (Eitan et al., 2010; Wainwright et al., 2010; Barakat et al., 2012; Orlando et al., 2012; Remlinger et al., 2012; Sullivan et al., 2012; Mirmalek-Sani et al., 2013a; Nichols et al., 2013; O’Neill et al., 2013; Park et al., 2013; Yagi et al., 2013; Gilpin et al., 2014; Weymann et al., 2014). The main challenge is the removal of xenoantigens – such as cell-surface galactose-alpha-l,3-galactose – which have thus far prevented successful pig-to-human transplantation (xenotransplantation). Although alpha-galactosyl epitopes are widely expressed on the surface of most mammalian cells, humans and apes lack these epitopes due to an inactivating mutation in the alpha-galactosyltransferase gene (Galili et al., 1984, 1985). Humans have natural antibodies against alpha-gal carbohydrates that account for ~1% of circulating immunoglobulins (Galili et al., 1988). Galactose-alpha-l,3-galactose expressed on the surface of pig endothelial cells is rapidly bound by these natural human antibodies (Cooper et al., 1994; Sandrin and McKenzie, 1994). The antigen–antibody complex activates complement (via the classical pathway) leading to vascular compromise and hyperacute rejection (Dalmasso et al., 1992). Thus, it is critical that the alpha-gal antigen be completely removed from pig organs in order to render the matrix immunologically inert.

Another issue with using pig organs as a scaffold source is the risk of transmission of porcine viruses such as PERV (porcine endogenous retrovirus), hepatitis E virus, porcine lymphotropic herpesviruses, porcine circoviruses, or porcine cytomegalovirus (Yoo and Giulivi, 2000; Scobie and Takeuchi, 2009). Various genetically modified pigs have been developed to reduce immunogenicity, but their organs can still be immunogenic (Le Bas-Bernardet et al., 2011; Scalea et al., 2012).

Because decellularization lyses cells and removes cell debris, it was hypothesized that this would eliminate xenoantigens as well as porcine viruses from pig organs. However, inadequate removal of xenoantigens has the potential to elicit an immune response upon implantation (Simon et al., 2003; Kasimir et al., 2006; Bastian et al., 2008; Cicha et al., 2011; Keane et al., 2012). In addition, there are conflicting reports about the transmission of PERV from porcine matrices (Kallenbach et al., 2004; Prabha and Verghese, 2008).

Many groups have demonstrated that porcine organs can be decellularized but have not assessed the removal of xenoantigens or porcine viruses. Nevertheless, recent reports have shown promise. Decellularization of sections of porcine livers with 0.1% SDS was reported to eliminate galactose-alpha-l,3-galactose as well as swine leukocyte antigens and PERV DNA (Park et al., 2013). Decellularized liver segments implanted subcutaneously into pigs had only mild immune cell infiltration in comparison to native implants, although all implants were completely degraded after 10 weeks *in vivo*. Another report utilizing decellularized porcine liver evaluated cytotoxicity *in vitro* using HepG2 (human hepatoblastoma) cells (Mirmalek-Sani et al., 2013b). The cells formed a dense layer on the edges of the scaffold with no signs of cytotoxicity. Mirmalek-Sani et al. went on to evaluate immunogenicity and biocompatibility by implanting acellular rat or pig liver scaffolds subcutaneously into rats and saw no evidence of an inflammatory response regardless of the scaffold source (allogenic or xenogenic). Systemic leukocyte counts were not statistically different between groups and no CD3+ T-cell activation was observed.

It is important that criteria for the evaluation of efficient removal of xenoantigens and porcine viruses be established and reported. Repeatable derivation of immunogen-reduced, biocompatible matrices from porcine organs will be critical to moving the field forward.

### Clinical application

The clinical application of decellularized (and recellularized) organ scaffolds is currently unknown territory. However, decellularized heart valves, blood vessels, and ECM from skin, bladder, pericardium, and small intestine submucosa has been evaluated for clinical application (Cebotari et al., 2011; Crapo et al., 2011; Dohmen, 2012; Moroni and Mirabella, 2014; Neumann et al., 2014). Decellularized skin ECM scaffolds may be one of the most predominately used acellular ECM matrices in clinical applications. There are numerous commercially available human acellular dermal grafts, for example: AlloDerm, LifeCell; FlexHD, Ethicon; Allopatch HD, Conmed; etc. [see Table 1 for a more complete list of commercially available acellular dermal matrix (ADMs)]. These types of acellular matrices are not only for the regeneration of skin but are also currently be used in surgical procedures such as abdominal wall reconstruction, treatment of complex trauma wounds, reconstruction of breast and areola tissue after mastectomies, and treatment of chronic scalp wounds (Craft and May, 2011; Janis et al., 2012; Seaman et al., 2012; Shitrit et al., 2014). Similar to ADMs, other decellularized ECM sheets have been successfully used in clinical application, such as porcine urinary bladder, which has been reported to help regenerate skeletal muscle in patients with volumetric muscle loss (Sicari et al., 2014).

**Table 1:** **Commercially available acellular dermal matrices**.

Commerical name	Manufacturer	Origin
AlloDerm	LifeCell	Human
FlexHD	Ethicon	Human
Allopatch HD	Conmed	Human
BellaDerm	Muscloskeletal Transplantation Foundation	Human
DermaMatrix	Synthes	Human
PerioDerm	Muscloskeletal Transplantation Foundation	Human
AlloMax	Davol	Human
DermACELL	LifeNet Health	Human
Glyaderm	Euro Skin Bank	Human

In regards to the use of commercially available decellularized matrices in a clinical setting in the United States, recellularization of ADM is often limited to cell migration from host tissues after implantation because most of these clinically used dermal matrices are available to the market due to a filing of “Pre-market Notifications” to the Food and Drug Administration (FDA) and are sold as medical devices. Hence, ADMs undergo a different FDA regulatory process than tissue and cell therapies (and eventually commercially available whole organ recellularized scaffolds, which will be regulated as a combination product). However, these widely used decellularized sheets may help pave the way for whole organ recellularized scaffolds by familiarizing patients and physicians with the concept of a decellularized matrix product. Additionally, ADM clinical use elucidates the human body’s ability to utilize and integrate natural, collagen-rich biological scaffolds for specific functions such as angiogenesis.

**Figure 2:**
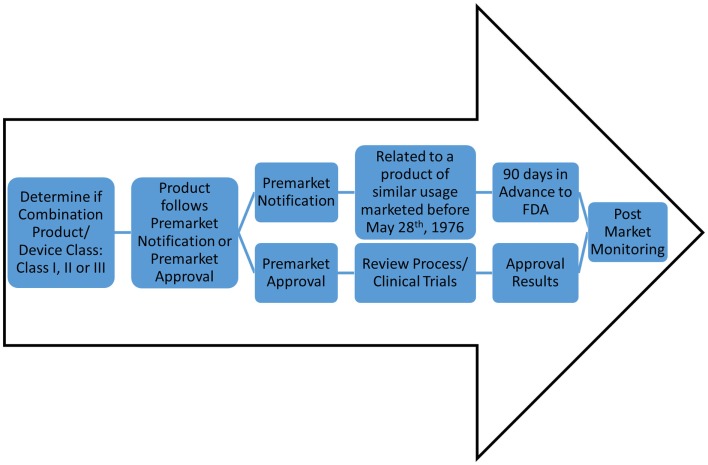
**Potential FDA regulatory routes of decellularized matrices**. For classification as a device, there are classes of device safety standards ranging from Class I (e.g., toothbrush) to Class III (e.g., pacemaker). However, an engineered organ will likely be classified as a combination product. Determination of product as Pre-market Notification or Pre-market Approval will allow for one of two routes: (1) 90 days notification to the FDA for market (for devices for similar use as products pre-dating May 28th, 1976), or (2) clinical trials and approval process; respectively. Both routes, once marketed and commercially available, will undergo stringent post-market monitoring.

Ultimately, the feasibility of engineered whole organs relies on successfully navigating the regulatory requirements of the FDA, both in the pre-market and post-market environment (Figure 2). Under the Code of Federal Regulations, regenerative medicine technologies fall into the category of combination product. The definition of a combination product is as follows: “A product comprises two or more regulated components, i.e., drug/device, biologic/device, drug/biologic, or drug/device/biologic, that are physically, chemically, or otherwise combined or mixed and produced as a single entity.” Combination products are regulated through two or more branches of the FDA, but are assigned to one office over another based on the primary means of action of said product. Ultimately, this designation will outline the path complex whole organs, such as lung or heart, will take toward market approval. According to the primary means of action of whole organs, the classification can fall either as a “biologic” or as a “medical device” and arguments can be made for either.

## Concluding Remarks

The critical components to successful recellularization are the utilization of (1) clinically relevant, renewable cell sources, (2) multiple seeding approaches, and (3) bioreactors with physiologically relevant organ culture conditions. Complete recellularization is a complex task, particularly for human-sized organs. A large number of cells encompassing a variety of cell types (parenchymal, vascular, and support) are required for sufficient recellularization. The ultimate goal is to use autologous cell sources to facilitate the production of patient-specific organs to improve the outcomes of transplantation. With these considerations in mind, the ideal cell type will be either stem or progenitor cells. Stem cells are efficiently expanded in culture, differentiate along multiple cell lineages, and isolated from autologous sources. Because of the ethical issues surrounding the use of ESCs, iPSCs or adult stem cells, such as ASCs or BMSCs may be viable options. These cell types have demonstrated potential for recellularization of the parenchyma and vasculature of organs. Although seeding methods will vary for each organ, the most promising results have been achieved when utilizing multiple seeding routes, several cell infusions, and a variety of cell types. Whole organ bioreactor culture must provide sufficient nutrient delivery to the developing organ along with other physiologically relevant stimuli, which is supported by reports of regenerated organs displaying functionality *in vivo*. Moving forward, optimizing revascularization is critical to successful organ regeneration and should be emphasized in future investigations. Lastly, the field has just begun to scale up decellularization, recellularization, and bioreactor technology to large animal models and human tissue. Concurrently, evaluation of immunogenicity and biocompatibility are of utmost importance to eventual FDA approval, commercial viability, and clinical application.

## Conflict of Interest Statement

The authors declare that the research was conducted in the absence of any commercial or financial relationships that could be construed as a potential conflict of interest.

## Supplementary Material

The Supplementary Material for this article can be found online at http://journal.frontiersin.org/article/10.3389/fbioe.2015.00043
