# Supplementary material for: A Review of Cellularization Strategies for Tissue Engineering of Whole Organs
Source: Front Bioeng Biotechnol. 2015 Mar 30;3:43. doi: 10.3389/fbioe.2015.00043 (PMC4378188; doi:10.3389/fbioe.2015.00043)
Supplement: Supplementary file 1 [file Table_1.PDF]

Supplemental Table 1. An Overview of the Kidney Recellularization Literature

Animal	Decell	Seeded Cells	Seeding Method	Culture Method	Additional Cues	Implanted	Outcome	Reference
Rat	Gravity-based perfusion at 100 mmHg into renal artery and ureter of 3% Triton X-100, Dnase, Triton again, then 4% SDS	2 x 10^{6} murine **ESCs**	Manual injection through either the **artery or ureter** followed by 24 hr static culture of whole organ in deep well; culture continued up to 14 days	Static organ culture led to significant apoptosis; so, automated perfusion system developed with pressure-relief to maintain 120/80mmHg	Authors noted that **no pro-differentiation agents** were added to media so that effect of kidney ECM alone one ESC differentiation could be assessed	No	95% of cells injected into artery retained; initially trapped in glomeruli but migrate into vasculature during extended culture; peritubular capillary location observed; cells die off to form lumens; at day 10, cells in vasculature of cortex appear reticular ; >50% of cells injected into ureter retained; not uniformly seeded (perhaps due to papillae); express Pax-2 and Ksp-cadherin	Ross *et al.* 2009
Rhesus macaque (slices)	Transverse sections of macaque kidney (**fetal, juvenile, or adult**) using 1% SDS at 4°C (37°C and Triton also tested)	**Fetal, juvenile, or adult macaque kidney explants**	Age-matched or non-matched explants layered on top of kidney scaffolds	Culture period of 5-7 days	Soaked in 70% ethanol and then pen/strep PBS for sterilization; scaffolds seeded with fetal fraction cells were cultured in FGF-rich media, EGM-2, or DMEM/F12 control media	No	Fetal, but not juvenile or adult, had cells infiltrate scaffolds; infiltration highest (0.3 mm) in fetal explant cells into fetal scaffold; infiltrating cells were either clustered vimentin+Pax2+ cytokeratin+ (epi-thelial) or individual vimentin+Pax2+ cytokeratin- (mesenchymal)	Nakayama *et al.* 2010 and Nakayama *et al.* 2011
		3rd trimester **fetal kidney cell fractions** (intact or dissociated)	500,000 dissociated cells per scaffold				Dissociated cells had no tubular infiltration but intact cells did; FGF-rich media enhanced tubular infiltration and maturation by intact cells while EGM-2 enhanced dissociated cell infiltration	
Rat	Gravity-based perfusion at 100 mmHg into renal artery and ureter of 3% Triton X-100, Dnase, Triton again, then 4% SDS	2 x 10^{6} murine **ESCs**	Manual injection into the **artery** followed by 24 hr static culture of whole organ in deep well; culture continued up to 14 days	Automated perfusion system with pressure-relief to maintain 120/80mmHg; sections of tissue were also cultured in traditional culture	**No pro-differentiation agents** were added to media	No	ESCs seeded into glomeruli and vasculature display lectin-specific (BsLB4) binding and expression of VEGFR2 denote endothelial differentiation; these cells only seen in vasculature and glomeruli, not tubular areas; mouse ESC-specific production of laminin β-1 chain and α1α2α1 collagen IV suggest **ECM remodeling and "murinization" of rat scaffold**	Ross *et al.* 2012

Porcine	Continuous perfusion through renal artery of SDS at 12 mL/min	5 x 10^5 murine hematopoietic support (MS1) immortalized **endothelial cell line**	4 mm sections of porcine kidney scaffold were cut and then statically seeded	Static culture of seeded slices	Scaffold sterilization by cobalt gamma irradiation (dose of 25 kGy over 6 hours)	No, but **acellular scaffold implanted into pig** for 2 weeks	MS1 cells adhered and lined matrix with no cytotoxic effects observed; blood flow throughout implanted acellular scaffold with no leak but after 2 weeks, massive thrombi and infiltration of inflammatory cells; fibrouse capsule (foreign-body response) encased one implant	Orlando *et al.* 2012
Porcine	0.25% SDS, 0.5% SDS, or 1% Triton X-100/0.1% NH_4OH perfused (0.75 L/h) into renal artery then DNase	1 x 10^5 primary **human renal cells** in renal cell medium (based on DMEM-HG)	5mm by 7mm biopsy of cortex was soaked in media for 3 days then seeded statically with cells	Static culture of seeded biopsies for 3-4 days	Sterilization by 10 kGy **gamma irradiation then storage for at least 6 months** at 37°C	No	All decell methods tested led to cell removal, DNA reduction, and ECM retention, but only 0.5% SDS reduced DNA to below 50 ng/mg; cells became necrotic after seeding on scaffolds decelled with Triton X-100; cells survived and grew along borders of scaffolds decelled using either SDS method	Sullivan *et al.* 2012
Rat	Renal Artery Perfusion of 1% SDS at constant pressure of 40 mmHg	50.67 ± 12.84 x10^6 Passage 8-10 **HUVECs** in 2 mL EGM-2	1 mL/min arterial flow followed by an o/n static incubation	Bioreactor-based whole-organ culture (3-5 days for HUVECs; up to 12 days for kidney cells)	Arterial perfusion culture at 1.5 mL/min	Yes, orthotopic into rat	Cells lined vessels throughout scaffold; no thrombi or hemorrhaging when implanted	Song *et al.* 2013
		60.71 ± 11.67 x10^6 Rat **neonatal kidney cells** in 2.5 mL REGM	Vacuum pressure gradient of -40 cmH_2O (seed via ureter) then o/n static incubation		Glucocorticoids and catecholamines added to media for cell maturation		Site-specific adhesion as well as polarity; recellularization of ~70% of glomeruli; some urine production *in vitro* and *in vivo*	
Rhesus macaque (slices) ***Lung* also assessed**	Kidney slices: 1% SDS at 4°C 10-14 days; Lung slices: 0.1% Triton at 4°C 20-24 days	5 x 10^5 **human ESCs** (WA09, passage 36)	Juvenile macaque kidney or lung sections of 8mm diameter statically seed in DMEM high glucose	Static culture for 8 days	**Lung slices were used for comparison of ECM-dependent ESC differentiation**; sterilization of scaffolds by 70% ethanol; scaffold conditioned media indicated presence of soluable factors	No	Cytokeratin+ epithelial pheno-types formed thick organized tubules in kidney; in lung, cells lined alveoli in thin networks with expression of surfactant proteins; in comparison to control hESCs, scaffolds drove differentiation (expressed kidney or lung genes) but no statistical differences between kidney vs lung scaffolds	Nakayama *et al.* 2013

Rat	Perfusion through renal artery at 0.4 mL/min with 1% SDS; pressure maintained at 62-107 mmHg	12 x10^6 R1 pluripotent **mouse ESCs** in 6 mL	Cells infused antegrade through renal artery via a syringe pump at 0.2 mL/min (6 mL total over 30 minutes)	Perfusion culture with recirculation of media for 24 or 72 hours	4 x 10^6 and 8 x 10^6 cells did not adequately repopulated the matrix; thus, 12 x 10^6 cells was used	No	97.5% of cells attached during seeding; 2.5% found in effluent during culture; even distribution in vascular network, peritubular capillaries, and glomeruli; 86% of glomeruli covered (>50% area) by cells; high proliferation and low apoptosis throughout culture; Oct-4 expression decreased while NCAM, Tie-2, and CD31 increased	Bonandrini *et al.* 2014
Rat	3 methods evaluated: 1% Triton X-100, 1% Triton and 0.1% SDS, or 0.05% EGTA with 0.02% trypsin and 1% Triton	5 x 10^6 **Human endothelial cells from iPSCs**	Injected by hand into the renal artery	3D scaffold in a petri dish for 36 hrs static	No	No, but acellular scaffold optho-topic implant into rat for 3 hours without clotting or bleeding	Endothelial cells outlined the branching structure of the vasculature adjacent to glomeruli	Caralt *et al.* 2015
		40 x 10^6 immortalized **human renal cortical tubular epithelial cells** (RCTEs) in 2 mL	Infused via bioreactor antegrade perfusion into renal artery at 25 mL/min for 15 minutes	Bioreactor culture continued at 4 mL/min for up to 7 days			RCTEs formed tubular structures; not found in arterioles suggesting translocation to parenchyma or peri-tubular regions; seeded ~50% of renal area; **lactate production increased, glucose consumption decreased during culture (suggests O^2 access limited in some areas)**	
Review: Katari *et al.* 2014 "Renal Bioengineering with Scaffolds Generated from Human Kidneys"								
Review: Figliuzzi *et al.* 2014 "Renal Bioengineering with Scaffolds Generated from Rat and Pig Kidneys"								
Review: Zambon *et al.* 2014 "Kidney Regeneration: Where we are and future perspectives"								
