# Supplementary material for: A Review of Cellularization Strategies for Tissue Engineering of Whole Organs
Source: Front Bioeng Biotechnol. 2015 Mar 30;3:43. doi: 10.3389/fbioe.2015.00043 (PMC4378188; doi:10.3389/fbioe.2015.00043)
Supplement: Supplementary file 2 [file Table_2.PDF]

Supplemental Table 2. An Overview of the Liver Recellularization Literature

Animal	Decell	Seeded Cells	Seeding Method	Culture Method	Additional Cues	Implanted	Outcome	Reference
Rat	Perfusion of 1, 2, and 3% Triton X-100 and 0.1% SDS at 5 mL/min	1 x 10^6 **WB344 rat liver progenitors** (cell line) in RPMI media	Infusion into the liver via the inferior vena cava	Not specified	Fetal bovine serum was perfused through the organ (to add growth factors presumably)	No	*Liver was decellularized *in situ* * Center of liver contained dense area of cells (by H&E) indicating that the cells were able to travel through the hepatic vasculature	Shupe *et al.* 2010
Rat	Frozen at -80°C for at least 4 hours then thawed at 4°C for perfusion via portal vein at 1 mL/min with 0.01% SDS, 0.1% SDS, then 1% SDS followed by 1% Triton	50 x 10^6 primary **rat hepatocytes** into the median lobe of the liver scaffold	Four steps (12.5 x10^6 cells each) at 10 minute intervals (superior to one infusion of 50 x 10^6)	5 d bioreactor culture with perfusion of media through portal vein	Sterilization with 0.1% peracetic acid; 2 hours after seeding scaffolds with 50 x 106 hepatocytes, scaffold was perfused with medium-diluted, heparin-containing rat blood for 24 hours	Yes, hetero-topically into a rat for 8 hours	>95% engraftment; well distributed in matrix after 4-5 days; <20% apoptotic in first 2 days; minimal cell death; seeding of large vessels and parenchyma (migrated from vasculature); retained functionality	Uygun *et al.* 2010
		200 x 10^6 primary **rat hepatocytes**	Four steps (50 x10^6 cells each) with 10 minute intervals	10 d bioreactor culture with perfusion			Scale-up seeding representing 20% of rat liver mass; maintained albumin, urea, and bile functions	
		40 x 10^6 cardiac **microvascular endothelial cells**	Portal vein infusion 24 hrs after seeding 50 x 10^6 hepatocytes	5 d bioreactor perfusion culture			Lined the vasculature surrounded by hepatocytes; remained viable; some unattached cells in lumen	
Ferret	Portal vein perfusion at 5 mL/min with 1% Triton and 0.1% NH_4OH	100 x 10^6 **mouse endothelial cells** (MS1)	Injected into **either portal vein or vena cava** then 2 hour static incubation	3 days of perfusion at 5 mL/min	Mouse, rat, and pig livers also decelled but not seeded; scaffold sterilized by gamma irradiation of 1.5 Mrad; hepatocyte seeded liver was perfused with fresh blood for platelet deposition experiment	No, but acellular scaffold ectopic transplant into rat had blood flow then clots after 1 hr	Seeding via vena cava deposits cells in the peri-central area; via portal vein deposits in periportal area; distribution of cells in large vessels and capillaries; anti-thrombotic function retained	Baptista *et al.* 2011
		70 x 10^6 **human fetal liver cells** with 30 x10^6 **HUVECs** co-seeded	Co-seeded through the portal vein over a 16 hour period at 3 mL/min perfusion flow rate	7 day bioreactor culture with perfusion at 0.5 mL/min			Hepatocyte markers detected in pattern indicating scaffold niches for bile duct and hepatocytes; endothelial markers stain cells in large vessels and capillaries; albumin and urea produced	
Mouse	*In situ* perfusion via portal vein at 5 mL/min with 1% SDS for 2 hrs, 1% Triton X-100 for 30 mins then PBS for 3 hrs	2-4 x 10^6 **immortalized human fetal hepatocytes** or 1-2 x 10^6 **primary human hepatocytes** in 1 mL of medium	Perfusion seeding	Cultured up to 1 week; or 24 hrs after seeding, 0.5 x 0.5 x 0.1 cm^3 section cut for implant	Matrigel encapsulation used as a control for comparison to decellularized liver matrix	Yes, into the omenta of immuno-deficient mice up to 8 weeks	Cells within decellularized liver matrix implants survived and functioned longer than cells directly injected into mice or implanted within Matrigel; in addition, host cells migrated into the liver matrix	Zhou *et al.* 2011

Rat	Frozen at -80°C for at least 24 hrs; perfusion via vena cava at 8 mL/min with 0.02% trypsin/ 0.05% EGTA then 3% Triton/0.05% EGTA	10-50 x 10^6 **mouse hepatocytes**	Five 200 µL injections into different hepatic lobes with a 27G needle/1 mL syringe	Bioreactor culture with perfusion at 2 mL/min	Sterilization with 0.1% peracetic acid and 4% ethanol; bioreactor culture media collected daily for detection of albumin	No	~13% engraftment; <1% proliferating; cell repopulate parenchyma near large vessels; lowest albumin production	Soto-Gutierrez *et al.* 2011
			Injected into bioreactor chamber media; circulation by constant perfusion				~69% engraftment; no proliferation; cell repopulate parenchyma near large vessels; lowest cytochrome P450 activity	
			Infused directly into liver perfusion circuit in steps with 10-15 min intervals				~86% engraftment; ~3% proliferating; cell repopulate parenchyma near large vessels; highest albumin production	
Rat	Pylic perfusion of 10 mM adenosine at a portal perfusion pressure of 25 mmHg then 1%SDS, 0.5% SDS, 0.25% SDS, and lastly, 1% Triton X-100	1 x 10^8 **rat hepatocytes spheroids** in 3 mL culture media	Direct infusion into the portal vein followed by 4 hr static incubation	2 hr perfusion culture at 1 mL/min prior to implantation	Scaffolds were coated with 9 layers of heparin using a layer-by-layer self-assembly technique (thromboresistant for 3h implantation)	**Yes, into rats with 90% hepa-tectomy**; 72 hrs *in vivo*	Seeded scaffolds had lattice-like, porous structures along the ECM architecture; after 72 hrs reperfusion, hepatocytes retained pyramidal shape and large round nuclei; organized into aggregates and vessel-like structures; had tight junctions and bile canaliculi with apical microvilli; express liver-related genes and synthesize albumin; slowed increase in blood ammonia levels (compared to control) and **prolonged life span from 16 to 72 hrs** (did not support long-term survival)	Bao *et al.* 2011
Porcine	Portal vein perfusion of 0.25% SDS then 48 hr incubation; perfusion of 0.5% SDS at 80 mmHg, next **collagen cross-linking, sterilization with 10% formalin**	3.5 x 10^8 **human fetal hepatocytes** and 1 x 10^9 **human fetal stellate cells**	Seeded into posterior segments of porcine liver matrices and then perfused for 3, 7, or 13 days	Bioreactor system based on "LifePort Kidney Trans-porter"; allows adjustment of air and CO_2 as well as infusion of fresh media depending on lactate and glucose concentrations	Scaffold sterilization (and cross-linking) by 10% formalin	No, but acellular scaffold transplant into pig showed perfusion for 2 hrs without leakage	Oxygen consumption (decrease of 13% from inflow through liver to outflow) during perfusion culture; glucose consumption and lactate and urea production increased during perfusion culture; retained ability to secrete albumin; more cells in proximal than distal area; >90% cell engraftment; from 3d to 13d, cells migrate from portal and hepatic vein into parenchyma; 40% hepatocytes proliferating, 20% stellate cells, 30% apoptosis, 20% AFP expression at 13d	Barakat *et al.* 2012

Rat	Froze -80°C overnight then thawed at 4°C; portal vein perfuse with water, 0.25% trypsin 0.038% EDTA, 1% NP-40, 1% saponin and 0.05% NaN_3, 0.5% Triton X-100, and PBS	1 x 10^6 **Bone marrow-derived MSCs** from GFP-transgenic C57BL/6 mice; **primary hepatocytes** from these mice were used as a control; cell concentration was 5 x 10^5 cells/mL	Scaffolds were frozen and **lyophilized**. Discs (10 mm x 3 mm) were cut and sterilized overnight with PBS containing antibiotics and antimycotics. Cells were dropped onto the **discs**; vacuum and aeration cycles were employed to enhance cell absorbtion	Static culture or dynamic culture in a perfusion chamber with recirculation of media at 0.5, 1, 2, 4, of 6 mL/min (4 mL/min was found to be optimal)	Hepatic differentiation of MSCs induced using differentiation (diff) media containing growth factors (including EGF, bFGF, HGF, nicotinamide, oncostatin, dexamethasone, and an insulin-transferrin-selenium mix)	No, but 3 x 10^6 **cells grown on decell scaffold were implanted** in the tail vein of CCl_4 fibrotic mice	By mRNA, IHC, flow cytometry, and functional analyses MSCs expressed liver markers when seeded on liver matrix; use of diff media enhanced MSC differentiation on liver matrix. Cells harvested from matrix transplanted into diseased mice were seen located around the portal tracts and interlobular connective tissue (up to 6 weeks after injection); increased survival and restoration of liver architecture	Ji *et al.* 2012
Rat	Perfusion at 0.54 mL/min of 4% Triton via portal vein, then Dnase and Rnase (0.5% and 1% Triton, and 1% SDS also tested); scaffold sterilized by 5% glutar-aldehyde then 70% ethanol	2-3.6 x 10^6 Human umbilical vein endothelial cells (**HUVECs**)	Perfusion seeding into portal vein at 0.5 mL/min	Static culture for 6 hrs then perfusion for 66 hours	Perfusion of 2.5 mL of heparinized rat blood for some experiments	No	Formed monolayer on internal surface of blood vessels; blood perfusion retained in endothelialized scaffold (not as many leaks as acellular matrix)	Shirakigawa *et al.* 2012 and Shirakigawa *et al.* 2013
		1 x 10^7 primary **rat hepatocytes**	Injected into **portal vein** then 30 min static incubation	Perfusion culture for 24 hours	None		Seeding through portal vein or hepatic vein "clogged up" the tubular structures with hepatocytes	
			Injected into **hepatic vein** then 30 min static incubation					
			Suspended in 0.24% collagen sol and injected via needle; 30 min incubation				Hepatocytes migrated from tubules to parenchyma; highest albumin production of the 3 hepatocyte seeding methods	
		1 x 10^6 **HepG2** cells and 2 x 10^6 **HUVECs**	HepG2 suspended in 0.25% collagen sol injected via needle then 1 hr for gelation, then HUVECs seeded via portal vein	Stationary culture for 6 hours then perfusion for 66 hours			HepG2 aggregates were localized near the re-endothelialized vascular tree	

Mouse	Portal vein perfusion of 1% SDS for 2 h then 1% Triton X-100 for 30 minutes at 5 mL/min	1 x 10^7 reversibly immortalized **mouse fetal hepatic progenitor cells**	Perfusion seeding through the portal vein	Static culture for 14 days	Cells were transduced with GFP or GFP and **epidermal growth factor (EGF)**	Yes, 0.5 cm^3 piece of seeded scaffold implanted into kidney capsule of athymic mouse for 10 days	*In vitro* - cells survive and proliferate for up to 2 weeks; *in vivo* - cells survive and proliferate for up to 10 days; EGF expression enhanced survival and proliferation as well as albumin expression; vascularization was also seen within scaffolds via staining for CD31	Xiaojun Wang *et al.* 2013
Porcine	Frozen at -80°C for 12+ hrs then thawed at 4°C for perfusion via portal vein at 1 mL/min with 0.01% SDS, 0.1% SDS, then 1% SDS, then 1% Triton	1 x 10^9 **primary porcine hepatocytes**	**Multistep infusion** of cells at 5-10 minute intervals; seeding was done in the context of the bioreactor	Perfusion culture at 4 mL/min with constant oxygenation (inflow partial oxygen tension of ~300 mmHg); up to 7 days in culture	Sterilization with 0.1% peracetic acid and ultraviolet irradiation for 1 hour prior to seeding	No	~92% cell viabillity; majority of cells attached to portal vein and large blood vessels after 24 hours but migrated to the parenchyma at day 4 and beyond; %apoptosis gradually increased throughout culture to ~48% at day 7; secreted albumin and urea but no difference in compison to controls; overall 74% grafting efficiency	Yagi *et al.* 2013
Porcine	Perfusion of increasing concentrationo f Triton (1%, 2%, then 3%) at 50 mL/min, then 0.1% SDS; rinsed with PBS	**HepG2** (hepato-blastoma) cells	15 mm **biopsy punches** were soaked in DMEM/F12 hepatocyte media then statically seeded	Static culture for up to 21 days	15 mm biopsy punches of scaffolds from rat or pig were sterilized prior to implantation with 1 Mrad (10 kGy) gamma irradiation	No, but rat or pig acellular scaffold biopsies implanted subcut-aneously into rats for 28 days to assess immuno-genicity	Statically seeded sections supported cells for up to 21 days with minimal apoptosis; dense cell layers noted on the surface of matrices with little cell infiltration into middle of matrix. **Acellular scaffolds**: no adverse host response to allogeneic (rat) or xenogeneic (pig) scaffolds implanted into rat for up to 28 day; host cells did migrate into scaffold but no inflammatory response observed	Mirmalek-Sani *et al.* 2013

Rat and Sheep	Two methods tested: (1) 1% Triton then 0.05% SDS or (2) only SDS; both used perfusion rate of 10 mL/min and constant shaking	No exogenously added cells	**"Body as a bioreactor"** concept; sheep or rat scaffold pieces implanted into rat for **"*in vivo*" recellularization**	$0.5\ cm^3$ scaffold pieces implanted into subhepatic area for 8 weeks	Decell method 1 determined to be better method (**implanted SDS only scaffolds had more immune reaction**)	Yes, to evaluate "*in vivo* " recell	Vessels with red blood cells were visualized in implanted scaffolds with diffuse inflammatory and fibroblast cells; rat scaffold faired better than sheep scaffold after rat implantation	Sabetkish *et al.* 2014
		18×10^6 **rat fetal hepatic cells** in 70 mL media	Two days of seeding through portal vein at 5 mL/min	**Media circulated 2 minutes every hour**, 15 day culture	Perfusion culture conducted on **shaker to prevent cell attachment to container wall**	No	*In vitro* cell seeding yielded better recellularization than "*in vivo* recellularization"; tissue structure was similar to normal liver with organized hepatocytes near vessels and in parenchyma	
Mouse	Froze at -80°C for 24h, then 1% Triton X-100 and 0.1% NH_4OH perfusion at 1 mL/min	**Mouse BMSCs** (P10-12)	Static experiments with 1-1.2 x 10^4 per cm^2; for implantation experiments, 50 x 10^6 cells in five 10 minute intervals were infused via portal vein the 1x1 mm piece excised	Static experiments assessed for hepatic differentiation over 4 weeks; **implants were placed into a incision in liver lobe of host**	Sterilization of scaffolds by gamma irradiation; **scaffolds used to investigate induction of MSCs to differentiate into hepatocytes**; **implants for treatment of CCl_4 liver damage**	Yes, as a **treatment method for fulminant hepatic failure in a mouse model**	Static experiments: presence of **liver scaffold significantly enhanced BMSC differentiation into hepatocyes** by all measures Implantation experiments: **significantly higher survival rate** when implanted with seeded liver scaffolds (**liver functions completely recovered after 7 days**; seeded scaffold engrafted and exhibited glycogen storage and albumin production)	Jiang *et al.* 2014
Rat	Froze -80°C for ≥24 hrs; perfusion of Trypsin, Triton X-100, and EGTA at 3 mL/min via the superior hepatic vena cava for 24 hours	**Primary rat hepatocytes** and **rat bone marrow-derived MSCs** at a ratio of 5:1; multiple cell numbers were evaluated (300, 100, or 50 million)	For co-seeding of hepatocytes and MSCs, 3 methods were evaluated: (1) hepatocytes seeded first, (2) MSCs seeded first, (3) cells seeded simultaneously; multi-step infusion similar to Soto-Gutierrez *et al.*	Perfusion culture up to 6 days	None	Yes, heparin infused scaffolds seeded with hepato-cytes alone or with MSCs implanted into rats for 1 hr	**Simultaneous seeding of hepatocytes and MSCs was the best seeding method**; seeding of higher cell numbers led to loss of scaffold integrity, cell retention and albumin production; hepatocytes lined the portal vein through the central vein similar to hepatic cords; MSCs were seen around portal area and intraluminal surface of vessels; CD31, VEGF, and integrin β1 expression by co-seeded cells; implantation led to perfusion but massive coagulation noted	Kadota *et al.* 2014
Review: Uygun *et al.* 2013 "Engineered Liver for Transplantation"								
Review: Caralt *et al.* 2014 "From the Stage of Liver Decellularized Matrix to the Multiple Cellular Actors and Bioreactor Special Effects"								
Review: Faulk *et al.* 2014 "Decellularization and Cell Seeding of Whole Liver Biologic Scaffolds Compose of Extracellular Matrix"								
