# Supplementary material for: A Review of Cellularization Strategies for Tissue Engineering of Whole Organs
Source: Front Bioeng Biotechnol. 2015 Mar 30;3:43. doi: 10.3389/fbioe.2015.00043 (PMC4378188; doi:10.3389/fbioe.2015.00043)
Supplement: Supplementary file 3 [file Table_3.PDF]

Supplemental Table 3. An Overview of the Lung Recellularization Literature

Animal	Decell	Seeded Cells	Seeding Method	Culture Method	Additional Cues	Implanted	Outcome	Reference
Rat	Retrograde pulmonary arterial perfusion with 0.1% SDS then 1% Triton X-100	$66.57 \pm 18.22 \times 10^6$ **HUVECs** in 20 mL media	Gravity perfusion through pulmonary artery and vein (4:1) with 1 hr static incubation	Continuous arterial perfusion at 10 to 15 mmHg; 9d culture	*In vitro* function indicated rat fetal lung cell + HUVEC seeded scaffolds had gas exchange, vital capacity, and compliance matching native lung; these scaffolds also contributed to gas exchange *in vivo* after orthotopic transplantation	Yes, ortho-topically into rats for **6 hrs without ventilator support; no airway bleeding, thrombi, or hema-toma; but pulmonaryedema observed**	Engraftment from pulmonary artery throughout branches to capillaries and pulmonary veins after 5 days bioreactor culture	Ott *et al.* 2010
		$91.25 \pm 31.72 \times 10^6$ **A549** alveoli basal epithelial cells in 15 mL	Gravity infusion through the trachea followed by 12 hour static incubation	Media ventilation for 5 days then dry ventilation (for *in vitro* functional gas exchange testing)			Human alveolar basal epithelial cell line (A549) seeded well but unregulated growth obliterated matrix after 5 days	
		308.57 ± 146.90 **rat fetal lung cells** and HUVECs					Engraftment from conducting airways to alveoli; **alveolary-capillary membranes intact** (0.2 to 4 µm); lack of ciliated cells but cuboidal cells observed	
Rat	Instillation at 37°C of 8 mM CHAPS, 1 M NaCl, and 25 mM EDTA into the airway and perfused at <20 mmHg into the vascualture; benzonase (90 U/mL) for nucelic acid digestion	~1×10^8 freshly isolated (no pre-plating) **neonatal lung cells** in ~10 mL	**Airway compartment** was fully inflated with cell suspension and allowed overnight static incubation for cell attachment	Perfusion at 1-5 mL/min or ventilation through negative pressure at 1 breath/min for 4-8 days for individual seeding; or 3-5 days for lung cell then seed endothelial cells with additional 24-36 hours	If liquid ventilation switched to **dry ventilation, increase in type I alveolar epithelial cells and ciliated columnar epithelial cells**; Human lung also decellularized as proof of concept; human scaffold seeded with **A549s** and **human cord blood endothelial progenitor cells**	Yes, ortho-topically into rats for 45 to 120 minutes with blood perfusion and gas exchange; **bleeding into air-ways** and partial inflation noted	Adherence in alveoli, medium and small airways; cells were highly proliferative in scaffold (not seen in standard tissue culture); media ventilation enhanced survival in distal portions and clearance of secretions; CCSP, aquaporin-5 (with dry ventilation), surfactant protein expression increased; **region-secific spatial distribution**	Petersen *et al.* 2010
		~3×10^7 **lung microvascular endothelial cells**	Instilled into vasculature at 3 mL/min with concurrent liquid media ventilation to facilitate diffuse seeding in **vascular compartment**				Vascular perfusion enhanced endothelial attachment and survival; CD-31 highly expressed and dispersed throughout matrix; **TEM showed tight junctions** (key to barrier function); however, bleeding into airways was noted when transplanted	

Mouse	0.1% Triton, 2% SDC, 1M NaCl, 30 ug/mL Dnase perfused and intra-tracheal	1-3 x 10^6 **mouse fetal lung cells** in 0.5 mL of small airway growth media	Injected into the trachea	7 day ventilation (at 180 breaths /min, 300 µL volume) in a bioreactor created from a 25 mL flask	None	No	Pro-surfactant protein C and cytokeratin 1 (markers of alveolar type II cells) present in cell throughout distal portion of the lung (alveolar areas); **no cells expressing CD11b, aquaporin-5, Clara cell secretory protein, CD31, or vimentin seen in lung**	Price *et al.* 2010
Rat	Frozen -70° C then fast thaw at 40°C (repeat 4x), rotating (2.5 rpm) bio-reactor with 1% SDS, then Dnase and RNase	2 x 10^6 **mouse ESCs** in 0.1 mL of PF-127 hydrogel	0.5 cm^3 piece used for recell; cells in gel injected into center of matrix piece (or Matrigel, Gelfoam, or collagen hydrogel); 5 min centrifugation at 800 rpm to spread cells in matrix	Constructs cultured staticaly for 24 hours before transferring to rotary bio-reactor with differentiation media for 6 d	Seeding on Matrigel, Gelfoam, or collagen hydrogel compared to acellular lung scaffold for mESC differnetiation (with lung differentiation media culture)	No	mESCs engrafted evenly throughout the acellular matrix piece but not Matrigel, Gelfoam, or collagen hydrogel; seeded matrix contained more cells and had less apoptosis; seeded **lung matrix increased expression of cytokeratin-18, pro-surfactant protein C, CD31 (lung markers)**	Cortiella *et al.* 2010
		2 x 10^6 **mouse ESCs** in 0.5 mL of PF-127 hydrogel	Whole trachea+lung recellularization via injection into main bronchi (1 x 10^6 to left and right); 800 rpm centrifugation	50 mL rotating bioreactor with lung diff. media for 14-21 days	Lung differentiation media used throughout culture		Shrinkage of the tissue; production of laminin and collagen IV (depleted by decellularization); PDGFR-α and cytokeratin-18 expression seen in scaffolds after 14 d; areas of CC10, αSMA, CD31, and pro-surfactant protein C expression resembled native lung; **measurable levels of surfactant protein A production**	
Rat	Retrograde pulmonary arterial perfusion with 0.1% SDS then 1% Triton X-100	**Rat fetal lung cells** and **HUVECs**	Lung cells seeded by gravity perfusion via trachea then 12 hr static incubation; 3 days later, gravity perfusion of HUVECs via pulmonary artery and vein (4:1) with 1 hr static incubation	Media ventilation started 24 hrs after lung cell seeding; dry negative pressure ventilation after 5 days	None	Yes, ortho-topically in nude athymic rats after left pneumo-nectomy	By improving graft preservation and adopting post-operative weaning protocol, **implants provided oxygenation for 7 days**; after 14 d, constructs appeared consolidated or restricted with increased cellularity (fibroblasts and macrophages); basophilic mucinous material in alveoli; emphysematous changes in central regions; no air leakage of bleeding; no detrimental effect on contralateral lung	Song *et al.* 2011

Mouse	0.1% Triton, 2% SDC, 1M NaCl, 30 ug/mL Dnase perfused and intra-tracheal	2 x 10^6 **mouse** bone marrow derived mesenchymal stem cells (**BMSCs**) in 3 mL basal media	Intra-tracheal inoculation then submerged in basal media overnight	Media was changed to fresh basal or small airway growth media next day; up to 28 d culture	**Small airway growth media** (SAGM) used in some experiments	No	Rounded (squamous) and flattened morphology in airways; no ciliated cells or epithelial-look cells; cell # increased in basal media and decrease in SAGM; only transient expression of TTF-1 lung marker; **osteoblast phenotype**	Daly *et al.* 2012
		**C10 mouse lung epithelial cells**					Different distribution than MSCs based on integrin binding; MSCS attached to collagen I and IV, laminin, and fibronectin	
Mouse	0.1% Triton, 2% SDC, 1M NaCl, Dnase perfused and intra-tracheal	2 x 10^6 mouse bone marrow derived MSCs (**BMSCs**) or **C10 mouse lung epithelial cells** in 3 mL media mixed with LMP agarose	Intratracheal injection then slicing (~1 mm) sections	Standard culture for up to 14 days	None	No	Despite differences in ECM and intracellular protein retention as well as structure and gelatinase activity, no major differences were observed when seeding scaffolds decellularized using one of three different protocols	Wallis *et al.* 2012
	0.1% SDS then 0.1% Triton X-100							
	8 mM CHAPS & 1M NaCl, 25 mM EDTA, Dnase, FBS							
Mouse	0.1% Triton, 2% SDC, 1M NaCl, 30 ug/mL Dnase perfused and intratracheal over a 24h period	4-8 x 10^6 **mouse ESCs** (E14tg2a) **induced to express TTF-1 and pro-SPC** (lung markers) in 400-500 µL	Intratracheal injection	Submersion in 4-5 mL of **differentiation media** and allowed to incubate for 24 hours	**Scaffolds were coated with Matrigel or collagen I** (or left uncoated) to assess cell seeding enhancement	Yes, sub-cutaneous implant in SCID/ beige mice for 14 days	Coating with Matrigel or collagen I increased the collagen content but did not ameliorate increased stiffness observed by mechanical testing; pre-differentiated mESCs seeded into uncoated or Matrigel-coated scaffolds expressed pro-SPC and TTF-1 but not in collagen coated scaffolds; after implantation, FOXJ1 expressed in uncoated and Matrigel coated but not collagen coated; in un-coated, neovascularization noted	Jensen *et al.* 2012

Human (disease model)	0.1% Triton, 2% SDC, 1M NaCl, 30 ug/mL Dnase perfused and intra-tracheal; series repeated 3x	1 x 10^5 normal **human lung fibroblasts** in 10 µL DMEM seeded dropwise onto slices	12 mm biopsy punch of scaffold embedded in 2% LMP agarose; 1 mm slices were cut; agarose removed by warming slices; cells seeded atop slice	30 minutes after seeding, slices were transferred to Teflon-coated plate; media changed every other day; cultured for 48 hours	Scaffold Sterilized prior to seeding using 0.18% peracetic acid and 4.8% ethanol for 20 minutes, then washed and stored in PBS at 4°C until seeded; **fibrotic scaffolds also used**	No	Cells migrated into the matrix; fibroblasts seeded onto normal lung matrices maintained phenotype defined by low α-SMA production whereas cells seeded onto fibrotic matrices displayed increase in production characteristic of myofibroblast differentiation	Booth *et al.* 2012
Rhesus macaque	0.1% Triton, 2% SDC, 1M NaCl, 30 ug/mL Dnase perfused and intra-tracheal	37.5 x 10^6 Rhesus macaque **BMSCs** or **ASCs** in 50 mL of a 1:1 mixture of α-MEM and 2% LMP agarose	Seeded **intra-tracheally** into a single lung lobe using a 60cc syringe; agarose allowed to solidify; ~1mm slices cut for static culture	Static culture of slices for 7 days; slices were inverted daily to better distribute media	None	No	Both BMSCs and ASCs attached to alveolar walls and air-conducting pathways; 20% of BMSCs and 25% of ASCs were proliferating at 7 days while 7.4% of BMSCs and 4% of ASCs were apoptosising; cells exhibit preferential binding based on specific ECM proteins	Bonvillain *et al.* 2012
Mouse	0.1% Triton, 2% SDC, 1M NaCl, 30 ug/mL Dnase perfused and intra-tracheal	1 x 106 mouse **BMSCs** or **C10 mouse lung epithelial cells** in 2 mL media-LMP agarose mix	Inoculations through the trachea; 30 minutes at 4°C to solidify agarose before slices cut	Standard culture in basal media for up to 28 days	Evaluation of the effect of delayed necropsy (72h), storage in PBS at 4°C with anti-biotics (3 or 6 months), and sterilization by gamma irradiation (60 Gy) or rinse in 0.1% peracetic acid in 4% ethanol	No	Despite structural and composition of the ECM, MSCs attached in the parenchyma (some restoration of normal appearance was noted after MSC seeding); no viable cells in 6 month storage matrix after 7-14 days in culture; **C10 cells were non-viable after 7-14 days on any of the manipulated matrices with significant increase in apoptosis**	Bonenfant *et al.* 2013
Mouse (disease model)	0.1% Triton, 2% SDC, 1M NaCl, 30 ug/mL Dnase perfused and intra-tracheal	1 x 10^6 mouse **BMSCs** in 1 mL IMDM with 1 mL LMP agarose	Cell-media-agarose mixture injected into trachea to seed left lobe; seeded lung was incubated for 30 minutes at 4°C to solidify agarose, then 1 mm slices were cut	Slices were cultured in 24 well dish with appropriate media; media was changed every other day; cultured up to 28 days	Matrices from rats with bleomyocin-induced fibrosis, elastase-induced emphysema (in young and aged mice), or old mice were also used	No	Cells mainly engraft in alveolar spaces; fibrotic lungs have areas where cells don't penetrate; BMSCs appear spindle-shaped; persist in scaffold for 28 days	Sokocevic *et al.* 2013
		1x 10^6 C10 **mouse lung epithelial cells** in 1 mL media with 1 mL LMP agarose					Cells mainly engraft in alveolar spaces; fibrotic lungs have areas where cells don't penetrate; C10 cells do not persist in emphysema scaffolds past 14 days	

Human and Porcine	Froze at -80° for >1 month, quick thawed at 45°C then 2% SDS into trachea and vascular tree, next 1% SDS from 100 mL/h up tp 500 mL/h for 7 days	**Mouse ESCs**<br>**Human fetal lung cells (HFLCs)**<br>**Pig BMSCs**<br>**Primary human alveolar epithelial type II cells (HAECs)**	2 x 10^{6} cells suspended in 0.1 mL of Pluronic-F127 hydrogel (15% solution in DMEM) seeded into the center of a 0.5 cm^{3} piece of pig or human scaffold, Matrigel, or Gelfoam; 5 min centrifugation at 100 *g*	24 hr static incubation followed by 7 day culture in a rotary bioreactor chamber	Scaffolds from pig or human were compared to Matrigel and Gelfoam	No	All four cell types adhered to pig scaffold while only a few cells attached to Matrigel or Gelfoam; viability and cellularity greatest for cells cultured on pig or human scaffolds than on Matrigel or Gelfoam after 7 days culture; no difference between cellularity of pig vs human scaffold; HAECs started clustered but spread out and expressed pro-SPC and some aquaporin-5;	Nichols *et al.* 2013
Rat	Instillation at 37°C of 8 mM CHAPS, 1 M NaCl, and 25 mM EDTA into the airway and perfused; benzonase (90 U/mL)	40 x 10^{6} **Human iPSC derived alveolar type II cells** (iPSC-AETII) in 3-5 mL media (small airway growth media)	Seeded into the airway then perfusion was immediately initiated at 1 mL/min	Maintained up to 7 days in bioreactor culture	None	No	iPSC-AETII engrafted throughout the alveoli of the distal lung; expression of classic AETII markers (pro-SPC, NKX2.1); iPSC-AETIIs in rat scaffold were pro-liferative with few apoptotic; some cells acquired flattened morphology and expressed T1α AETI marker (but did not express NKX2.1); T1α+ cells increased while pro-SPC decreased	Ghaedi *et al.* 2013
Human (slices 0.6 mm thick)	0.1% Triton, 2% SDC, 1M NaCl, 30 ug/mL Dnase incubation & agitation	3 x 10^{5} **iPSC-AETII** (or **native human AETII** for comparison)	Seeded onto decellularized human lung slice in small airway growth media	1 week static culture			iPSC-AETIIs adhered to rat and human scaffold slices throughout alveoli with AETII morphology and marker expression; some cells acquired AETI morphology and expression of T1α	
Human and Porcine (slices)	Froze at -80° submerge in 8 mM CHAPS (1.8 mM SDS was tested as well as 3% Tween 20 with 4% SDC and a wash with 0.1% per-acetic acid)	2.5 x 10^{4} cell/mL of **human lung fibroblasts** (hMRC-5s), **human small airway epithelial cells** (hSAECs), and **human adipose-derived mesenchymal stem cells** (hASCs)	Seeded onto 7 mm scaffold discs	Cultured for 7 days	Human and porcine decell lung were largely similar in retention of ECM (except elastin was better retained in porcine); human matrix was stiffer than porcine matrix	No	CHAPS decellularization was optimal method; hMRC-5s, hSAECs, and hASCs attached and proliferated at comparable rate over a 7-day culture period on CHAPS decellularized lung matrix; the three cell types had comparable metabolic rates, but hSAECs were more metabolically active on human scaffold than on porcine scaffold, perhaps due species-specific cues	O'Neill *et al.* 2013

Rhesus macaque	0.1% Triton, 2% SDC, 1M NaCl, 30 ug/mL Dnase perfused and intra-tracheal	1 x 10^6 Rhesus macaque **BMSCs** per mL in 50 mL	Within bioreactor, cells injected into trachea with syringe; o/n static incubation	14 d bioreactor culture; media instilled 1 breath/ 2 min	None	No	BMSCs lined alveolar walls; lumen unobstructed; large airways also contained cells (that appeared as a squamous-like cell monolayer)	Bonvillain *et al.* 2013
		8 x 10^4 Rhesus macaque **microvascular endothelial cells** per mL	Perfusion seeded within bioreactor at ~10 mL/min followed by ~4-6 h static incubation	5 d bioreactor culture; media perfusion at 5 mL/min	Stir bar added to seeding reservoir during seeding		Microvascular endothelial cells lined the small vasculature; some cells appeared to attach across lumen while others had clear lumen	
Rat	Instillation at 37°C of 8 mM CHAPS, 1 M NaCl, and 25 mM EDTA into the airway and perfused at <20 mmHg into the vascualture; benzonase (90 U/mL) for nucelic acid digestion	2.5-10 x 10^6 human **bone marrow-derived MSCs**	Injected as a bolus through the trachea into the right upper lobe	7 day bioreactor culture in small airway growth media (SAGM) with perfusion via the pulmonary artery at 1 mL/min	SAGM was used rather than DMEM because scaffold seeding and culture in **DMEM led exclusively to myofibroblasts** (αSMA expression) ; as a control, **seeding of bone marrow MSCs on decellularized liver did not yield pulmonary lineages**	No	Cuboidal appearance of attached cells throughout alveolar matrix with no cells in proximal airways; 65-70% express pro-SPC. Cytokeratin-5 expressed by some cells; no cells expressed CCSP; **produced surfactant and lamellar bodies and secretory vesicles**	Mendez *et al.* 2014
		2.5-10 x 10^6 human **adipose-derived MSCs**					Engrafted througout matrix but had **propensity to repopulate the proximal and small airways**; positive for pro-SPC and CCSP but not cytokeratin-5 (unlike BMSCs); **produced significantly more SPC (visible oil droplets) than BMSCs**	
Human (disease model)	0.1% Triton, 2% SDC, 1M NaCl, 30 ug/mL Dnase perfused (at 2L/min) and intra-tracheal on shaker table	**Human bronchial epithelial cells** in DMEM/F-12	2 cm^3 pieces of scaffold were coated with 2.5% sodium alginate and cross-linked with 3% CaCl to form calcium alginate hydrogel; small airways/blood vessels were innoculated with biologically relevent cell type	Cell seeding was followed by o/n static incubation, then slices were cut for static culture in 24 well dish with 2 mL media per well; cultured up to 1 month	Lungs from individuals with emphysema were used; scaffolds were sterilized using 0.1% peracetic acid in 4% ethanol	No	Localized mainly to alveolar spaces/parenchyma; cells attached (flattened) in normal lung but looked round in emphysema lung; unattached cells undergo anoikis; HBEs and hBMSCs persist up to 21 days; HLFs up to 28 days; no cells past 7 days in emphysema lung	Wagner *et al.* 2014
		**Human lung fibroblasts** in DMEM/F-12						
		**Human BMSCs** in MEM-EBSS						
		CBF (**endothelial colony forming cells**) in cEGM-2					Localized primarily to blood vessels; persisted up to 21 days in normal and 7 days in emphysema	

Rat (disease model)	0.1% Triton, 2% SDC, 1M NaCl, 30 ug/mL Dnase perfused and intra-tracheal	5-8 x 10^6 rat **ASCs** in to 8-10 mL of a 1:1 mixture of α-MEM and 2% LMP agarose	Seeded either **intra-tracheally or vascularly** using a 10cc syringe; agarose allowed to solidify; ~1mm slices cut for static culture	Static culture of slices for up to 14 days; slices were inverted daily to better distribute media	In addition to normal rat lung scaffolds, **scaffolds from rats with pulmonary hypertension were seeded as well**	No	Cells attached to alveolar walls and bronchioles (displaying varying morphologies); cells lined vascular walls; % of proliferating cells decreased during culture time (no change in apoptosis); cells appeared to produce elastin	Scarritt *et al.* 2014
Rat	4% SDC and 3% Triton in rotary (5rpm) carousel hybridization oven; con-centration gradually decreased to 0.5%, then DNA-away and Dnase	1 x 10^7 **mouse ESCs**	Gravitational inoculation into the trachea followed by 1 hr incubation at 37°C	No culture period; lungs harvested after incubation for analyses	**Matrices coated with laminin and fibronectin** (from A549 conditioned media) to enhance mESC attachment (based on integrin profiling, mESCs can attach to laminin and fibronectin but not collagen I or IV)	No	Coating of the matrix by instillation of conditioned media from A549 cells enhanced the number of cells reatined in the matrix after seeding by 2.3-fold; coating aso facilitated mESC uniform distribution along the cranial-caudal axis	Lecht *et al.* 2014
Rat, Porcine, and Human (slices)	Three methods tested: (1) 0.1% SDS (2) 2% SDC of (3) 8mM CHAPS	5 x 10^5 **Small airway epithelial cells** (SAECs), **pulmonary alveolar epithelial cells** (PAECs), or **HUVECs**	Seeded directly on top of lung matrix slices; **PAECs (500 x 10^6) were also seeded into the upper right lobe of a human lung** then statically incubated for 3 hours	Slices cultured for 5 days in small airway growth media, alveolar epi-thelial media, or endothelial growth media; human lung cultured for 96 hrs with at 30 mmHg	None	No	All cell types used were highly viable; Cell-matrix interaction via integrins α2β1 and α3β1 (for collagen I/IV and laminin 5/10/11, respectively); matrices were biocompatible; culture of seeded lobe of human lung indicated no tissue damage or contamination with intact vasculature; cells were well distributed and retained; appearance of flattened epithelial phenotypes	Gilpin *et al.* 2014
Rat	Pulsatile flow of 0.05% SDS via trachea, 38°C, 0.05 mL/min	2 x 10^6 **neonatal lung cells** in 70 mL DMEM followed by another 20 x 10^6 cells 2 days later	Static seeding for 18 hours in petri dishes containing cells; then bioreactor seeding (2 minutes every hour) tracheal flow at 5 mL/min over 17 days	No additional culture period used, just bioreactor-based seeding over 2.5 weeks	Shaker was used during bioreactor to prevent cell adherence to the container wall	No	Cells were distributed throughout the scaffolds with some cells staining positive for TTF-1; mucin secretion was detected in some areas; maintenance of collagen in seeded lung was also noted	Kajbafzadeh *et al.* 2014
Review: Calle et al. 2013 "Strategies for Whole Lung Engineering:								
Review: Wagner et al. 2013 "Can Stem Cells be Used to Generate New Lungs? Ex Vivo Lung Bioengineering with Decellularized Whole Lung Scaffolds"								
