# Supplementary material for: A Review of Cellularization Strategies for Tissue Engineering of Whole Organs
Source: Front Bioeng Biotechnol. 2015 Mar 30;3:43. doi: 10.3389/fbioe.2015.00043 (PMC4378188; doi:10.3389/fbioe.2015.00043)
Supplement: Supplementary file 4 [file Table_4.PDF]

Supplemental Table 4. An Overview of the Heart Recellularization Literature

Animal	Decell	Seeded Cells	Seeding Method	Culture Method	Additional Cues	Implanted	Outcome	Reference
Rat	Retrograde coronary perfusion at pressure of 77.4 mmHg with 10 µM adenosine, 1% SDS, then 1% Triton X-100	50-70 x 10^6 **neonatal cardiac cells** in PBS	5 injections (200 uL each) into anterior left ventricle with 27G needle and 1cc syringe	Bioreactor culture: 20 mL/min atrial flow; 6 mL/min coronary flow; up to 28 day culture	Electrical stimuli with 10 ms pulses of 5V starting 24 hrs after seeding	No, but decell scaffold implanted to show perfusion and valve function	High cell density near injection sites; viability >95%; sarcomeric α-actin and cardiac myosin heavy chain expression; some connexin-43 and vWF; immature cross-striated contractile fibers at 8-10 d; electric/contractile responses	Ott *et al.* 2008
		2 x 10^7 **rat aortic endothelial cells**	Direct infusion into aorta then static 45 minute period	Bioreactor perfusion culture; 1 wk	None		Single layers in large and small coronary vessels; ventricular cavities also reendothelialized	
Porcine (sheets)	Froze (-80°C) then aortic perfusion of 0.02% trypsin, 0.05% EDTA, 0.05% NaN_3 solution, then 4% SDC	Hamburger-Hamilton Stage 31 (day 7) White leghorn **chicken embryonic cardiomyocytes**	Lyophilized sheets of porcine cardiac ECM seeded with 500,000 cells/cm^2 on the luminal side	Cultured for 4 days in DMEM containing 1% chick embryo extract, 10% FBS, and 1% pen/strept	Scaffolds sterilized by perfusion of 0.1% peracetic acid/4% ethanol; terminal sterilization prior to seeding with ethylene oxide	No	Cardiomyocytes displayed organized sarcomere structure (striations of the α-actinin fibers)	Wainwright *et al.* 2010
Porcine (3 mm thick slices)	1.1% NaCl - 0.02% EDTA, then 0.7% NaCl - 0.02% EDTA, then 1% Triton X-100 and 0.1% NH_4OH, then 70% ethanol, then lyophilized	Sheep cardiac **fibroblasts** in DMEM-high	After lyophilized scaffolds were rehydrated in media, 5 x 10^5 cells were pipetted slowly onto a 10 x 10 mm scaffold	Static incubation of seeded slice for up to 6 weeks	None	No	Scaffolds shrunk into densely populated spheroids; organized and secreted fibers; high cell viability; matrix remodeling (increased expression of collagen, GAG, MMP2, and TIMP1)	Eitan *et al.* 2010
		Neonatal rat cardiac **myocytes** in DMEM-high					High cell viability; f-actin, cardiac connexin-43, α-actinin, and troponin I expression; gap junctions; elongated morphology; spontaneous pulsatile contractions after 2-3 days; persisted for 4 weeks	
		Rat **BMSCs** in DMEM-low					High cell viability	

Rat	Compared 4 methods including Ott and Badylak protocols as well as a combinationm ethod	**C2C12 myoblasts**	Seeded onto the surface of scaffolds	24 hour culture	None	No	Software-controlled automatic coronary perfusion-based decellularization methods led to variations in ECM retention but C2C12 myoblasts were able to reseed all scaffolds regardless of decellularization method used; none of the methods were ideal	Akhyari *et al.* 2011
Mouse	Aortic perfusion of 1% SDS overnight then 1% Triton X-100 for 1 hour	HUES-7 **human ESCs**<br>**Human mesendo-dermal cells** (differentiated from ESCs)	3 x 10^{6} cells in 100 uL media manually injected into aorta	Static culture submerged in conditioned media (from rat myocardial H9c2 cells) for up to 14 days	None	Yes, sub-cutaneous implant of 14-day cultured heart into SCID mice up to 6 weeks	Loss of stem markers; gain of Nkx2.5 and cTnT (expression similar between hESCs and hMECs); myosin chains - Myh6 expressed only by hESC while Myl2 and Myl7 only expressed by hMECs; when implanted, more cells and better vascularization of hMEC hearts; some endothelial differentiation (CD31) exclusive to vasculature; no beating (common for cardiomyocytes) observed	Ng *et al.* 2011
Rat	Retrograde coronary perfusion at 2 mL/min with 1% SDS followed by **cryostorage** in 10% DMSO in PBS	2 x 10^{7} **canine blood outgrowth endothelial cells** in 2 mL MCDB with endothelial growth media-2 singlequot	Injected into vasculature and walls of heart with 18G needle; static culture for 45 minutes	Culture in a modified spinner flask with heart partially submerged; media recirculation at 3 mL/min; 9 day culture	**Scaffolds stored at -80°C for up to 1 yr**; thawed then perfused with 1% Triton X-100 then 6.7 U/mL benzonase, then 1.8% skim milk solution; sterilized with 0.1% peracetic acid and 4% EtOH	No	Cryopreserved scaffolds seeded with endothelial cells showed adhesion throughout the tissue after 9 days of culture; clusters of cells suggest proliferation; cells were well spread and formed multiple focal adhesions; cells were viable and ECM was not cytotoxic	Crawford *et al.* 2012
Rat	Akhyari *et al.* combination method (details described in Aubin *et al.* 2013)	1 x 10^{6} **Murine C2C12 myoblasts** in 1 mL of low-glucose l-glutamax DMEM	Injected 100 uL containing 100,000 cells 10x across left ventricular wall scaffold; 24 hr later, stretch initiated; 96 hour culture	Bioreactor culture with coronary per-fusion at 70 mmHg and left **ventricular balloon stretch**	Prior to seeding, scaffold was perfused with fetal calf serum to load scaffold with growth factors	No	Mechanical stimulation by perfusion and controlled, 3D left ventricular stretch in a custom bioreactor led to increase in spatial orientation to form aligned network (in comparison to static controls)	Hülsmann *et al.* 2013

Porcine (20 x 20 x 3 mm patch)	In rotating bioreactor, 0.1% SDS, 0.01% tryp-sin, 1 mM PMSF, 20 µg/mL Rnase A, 0.2 mg/mL Dnase for 2.5 weeks; daily 10 min ultrasonic treatment	1 x 10^6 rat **MSCs** in 1 mL L-DMEM	Scaffold mounted between a fixed and a movable clamp then sterilized for 2 hr with 70% ethanol followed by UV irradiation for 20 minutes; cells injected into scaffold sample using syringe at nine spots (~100 uL/spot, 3 x 3 array)	Cultured in azacytidine differentiation media with cardiac myocyte growth supplement for up to 4 days	20% strain stimulation and/or 5V electrical stimulation (frequency of 1 Hz) were used to assess the effect of these stimuli on MSC-based cardiac tissue engineering	No	Mechanical/electrical stimulation increased cell density and cell survival (over static control); cell alignment was also observed when strain was involved but not electricity; scaffolds with both forms of stimulation had superior density, alignment, morphology, and increased cardiomyocyte differentiation as well as better biaxial mechanical behavior	Bo Wang *et al.* 2013
Mouse	Froze (-80°C), perfusion of 0.02% trypsin, 0.05% EDTA, 0.05% NaN_3 solution, then 1% SDS/ 0.05% NaN^3, then 3% Triton X-100/0.05% EDTA/0.05% NaN^3, 4% deoxycholic acid	1 x 10^7 day 6 dissociated **iPSC** embryoid body cells (with cardiomyocyte, smooth muscle, and endothelial cell differ-entiation) in basic differ-entiation media; **human RUES2 embryonic stem cell derived cardiovascular progenitors**	Cells were suspended in basic differentiation media containing gluatmine, monothioglycerol, ascorbic acid, transferrin, and pen/strept; delivered into the scaffold via cannulated aorta; culture for up to 16 days	**Did not employ continuous perfusion** since it washed out most of the cells within 5 days; **used periodic perfusion** (30 minutes every 8 hours) with growth factors (**VEGF, bFGF, DKK1**) to facilitate differentiation	Sterilization by 0.1% peracetic acid/4% ethanol; seeded scaffolds were tested for **drug response** using isoproterenol β1-adrenergic agonist and E4031 blocker of the rapid component of the delayed rectifying K^+ current (elicits response similar to polymorphic ventricular arrhythmias)	No	iPSC-derived myocyte progenitors repopulated matrix; exhibited spontaneous contraction at 20 days; irregular wave morphology (by EKG) showed lack of controlled conduction system; sporadic electrical cell-cell coupling observed; monolayer on endo-cardium; muscle-like and vessel-like (with branching) structures form; cardiovascular marker expression; drug responsive (isoproterenol increased contraction rate; irregular Ca^{2+} amplitude from E4031); higher contractile force from higher $[Ca^{2+}]_{out}$	Lu *et al.* 2013
Human (300 µm section)	Multiple methods; optimal method = lysis buffer for 2 hr, then 0.5% SDS for 6 hrs shaking, PBS washes, FBS 3 hr 37°C	0.5 x 10^6 **human umbilical cord blood MSCs** per cm^2 in DMEM	Pieces of ECM cut and cells seeded on top; cells that didn't attach often attached to tissue culture plastic instead; seeding via needle wasn't better method	Normal static culture up to 3 weeks	Uncoated, native cardiac ECM scaffolds used for seeding	No	MSCs infiltrated scaffold over time; viability/metabolic activity were higher when MSCs were grown in presence of scaffold	Oberwallner *et al.* 2013
		Murine **cardiomyocytes from iPSCs** in DMEM high			Scaffolds coated with 2.5 µg/mL fibronectin were used		iPSC-cardiomyocytes attached less fervently than MSCs and did not infiltrate matrix (due to low proliferative/migratory ability)	
		Murine **neonatal cardiomyocytes**			Fibronectin coated and uncoated scaffolds used		Attached well with or without fibronectin coating; synchronous beating observed	

Rat	Retrograde coronary perfusion of 1% SDS at 80 mmHg and 1% Triton X-100	2-4 x 10^7 **Rat aortic endothelial cells** (RAECs) in MCDB-131 media; 1.3 x 10^8 **rat neonatal cardiac cells** seeded into left ventricle by direct injection for functional evaluation	**Methods evaluated**: (1) 2 x 10^7 cells through aorta then 1 hr static incubation before aortic perfusion resumed; (2) 2 or 4 x 10^7 cells into brachio-cephalic artery (BA) during perfusion; (3) inferior vena cava (IVC) infusion, resumed perfusion then seeded via BA (2 x 10^7 per route); (4) 3 x 10^7 cells by IVC route only	Bioreactor culture with continuous aortic perfusion; **flow rate gradually increased from 1 to 3 mL/min** over 3 days; culture period of 7 days	**Thrombogenicity evaluated** on last day of culture by perfusion of human α-thrombin and human protein C through the seeded scaffolds for 45 minutes at 1 mL/min	Yes, BA seeded lungs were trans-planted hetero-topically into rat for 7 days	**Dual IVC+BA route yielded highest cellularity**; by BA or dual seeding - uniform distribution of cells throughout matrix though BA seeded mostly left ventricle and IVC seeded right ventricle; flattened morphology and no vessel occlusion; cells were viable, metabolically active, and functional (by PCNA, eNOS, and vWF expression as well as **thrombomodulin and thrombin-mediated protein C activity**); tranplanted lung had less clotting than acellular control; cardiac cell seeding and BA endothelialization improved beating and cellularity	Robertson *et al.* 2014
Porcine	Constant antegrade coronary perfusion of 37°C 4% SDS at 2 L/min, 100 mmHg, for 12 hours, then PBS	5-6 x 10^6 passage 3-6 human umbilical vein endothelial cells (**HUVECs**)	Infused into coronary arteries via aorta; perfusion was suspeneded for 60 minutes to facilitate cell attachment	Specialized bioreactor culture; media changed every 12 hrs; max flow of 3.5 L/min; up to 3 week culture	Segments of seeded myocardium used for measurement of electrical activity	No	Layer of PECAM-1^+, cobblestone-like HUVECs layered large and small coronary vessels; incomplete recellularization (attributed to **loss of many cells in the effluent**)	Weymann *et al.* 2014
		8-9 x 10^6 neonatal rat **cardiomyocytes**	5 injections (8-10 mm deep) of 200 uL each into left ventricle				~50% cellularity at injection sites; less seeding in distal areas; **electrical activity** (200 mV)	
Review: Momtahan *et al.* 2014 "Strategies and processes to decellularize and recellularize hearts to generate functional organs and reduce the risk of thrombosis"								
