# Supplementary material for: A Review of Cellularization Strategies for Tissue Engineering of Whole Organs
Source: Front Bioeng Biotechnol. 2015 Mar 30;3:43. doi: 10.3389/fbioe.2015.00043 (PMC4378188; doi:10.3389/fbioe.2015.00043)
Supplement: Supplementary file 5 [file Table_5.PDF]

Supplemental Table 5. An Overview of the Pancreas Recellularization Literature

Animal	Decell	Seeded Cells	Seeding Method	Culture Method	Additional Cues	Implanted	Outcome	Reference
Rat	Slices were processed with 4% SDC, 2000 kU Dnase, 1 M NaCl	50 freshly isolated **rat islets** per scaffold slice in RPMI media	Cells were seeded atop matrix	Static culture for up to 42 days	Decellularized liver slices used as a control; seeded pancreas alone or inside a PVA/PEG tube was tested	Yes, 5 matrices in one PVA/PEG tube, 3 tubes per rat	Seeded pancreas matrix showed preserved insulin secretion up to 6 weeks; islet function impaired on liver matrix; when in PVA/PEG tubes and implanted into diabetic rats, significant reduction of blood-glucose levels	De Carlo *et al.* 2010
Rat	Perfusion-based decell	200 x 10^6 **Human islet cells** and 10 x 10^6 **MSCs**	NA	Bioreactor culture	NA	Yes	Preservation of viability & insulin response to glucose challenge after bioreactor culture; islet cells and MSCs were in close proximity; MSCs home to islet cell niche	Conrad *et al.* 2010 ABSTRACT
Porcine	Perfuse with 1% Triton X-100/0.1% ammonium hydroxide for 24 h at 0.75 L/h via pancreatic duct and superior mesenteric vein	**Human amniotic fluid-derived stem cells** (hAFSCs) in Chang media	Either hAFSCs or porcine pancreatic islets were seeded atop 7 mm biopsy punches of porcine pancreas scaffold in appropriate media	Static culture for up to 7 days	None	No	After 48 hours, hAFSCs engrafted on the surface of pancreas ECM; after 7 days, cells migrated into scaffold; 2-fold increase in cell number from 3 to 7 days in culture	Mirmalek-Sani *et al.* 2013
		Freshly-isolated pig pancreatic **islet cells** in modified CMRL-based serum-free media					Metabolic rate increased 2-fold from 3 to 7 days in culture; insulin secretion after 3 days on scaffold was significantly higher than in traditional cell-culture controls	
Mouse	Retrograde perfusion of 0.5% SDS, then 1% Triton X-100, benzonase, lastly FBS at 8 mL/min	30 x 10^6 **MIN-6** β cells (passage 19-26) in 3 mL DMEM only or 30 x 10^6 MIN-6 cells and then 30 x 10^6 **AR42J** acinar cells (passage 18-25) in 3 mL F-12K media	MIN-6 cells seeded into the hepatic portal vein via retrograde gravity perfusion in three 1 mL steps with 20 min static incubation between steps; AR42J seeded into pancreatic duct (after MIN-6 seeding) using the same method	After 2 h static incubation, seeded scaffold was rinsed through both routes; submerged in media for 5 day static culture with perfusion feeding once daily	Dual-seeded pancreas used anatomical proximity of the cells to their physiologically relevant secretory locations (endocrine β cells near vasculature while exocrine acinar cells in ductal system)	No, but acellular scaffold was; showed blood vessel infiltration and no foreign body response	MIN-6 cells attached in luminal spaces near parenchymal region and larger blood vessels with <18% apoptosis and retention of insulin expression; AR42J cells lined tubular ductal spaces with retension of amylase expression; no co-localization of the two cells types were observed (meaning the vascular and ductal compartments were intact)	Goh *et al.* 2013

Review: Ross et al. 2013 "Regeneration and bioengineering of transplantable abdominal organs"
